# Dual roles of mTORC1-dependent activation of the ubiquitin-proteasome system in muscle proteostasis

**DOI:** 10.1038/s42003-022-04097-y

**Published:** 2022-10-27

**Authors:** Marco S. Kaiser, Giulia Milan, Daniel J. Ham, Shuo Lin, Filippo Oliveri, Kathrin Chojnowska, Lionel A. Tintignac, Nitish Mittal, Christian E. Zimmerli, David J. Glass, Mihaela Zavolan, Markus A. Rüegg

**Affiliations:** 1 Biozentrum, University of Basel, Basel, Switzerland; 2 Novartis Institutes for Biomedical Research, Cambridge, MA USA; 3 Present Address: BIOREBA AG, Christoph Merian-Ring 7, 4153 Reinach, Switzerland; 4 Present Address: Department of Biomedicine, University of Basel, Hebelstrasse 20, 4031 Basel, Switzerland; 5 Present Address: AstraZeneca AG, Neuhofstrasse 34, 6340 Baar, Switzerland; 6 Present Address: Neuromuscular Research Group, Departments of Neurology and Biomedicine, University of Basel, University Hospital Basel, Basel, Switzerland; 7 Present Address: Department of Molecular Sociology, Max Planck Institute of Biophysics, Max-von-Laue-Straße 3, 60438 Frankfurt am Main, Germany; 8 Present Address: Regeneron Pharmaceuticals, Tarrytown, NY USA

**Keywords:** Ageing, Metabolism

## Abstract

Muscle size is controlled by the PI3K-PKB/Akt-mTORC1-FoxO pathway, which integrates signals from growth factors, energy and amino acids to activate protein synthesis and inhibit protein breakdown. While mTORC1 activity is necessary for PKB/Akt-induced muscle hypertrophy, its constant activation alone induces muscle atrophy. Here we show that this paradox is based on mTORC1 activity promoting protein breakdown through the ubiquitin-proteasome system (UPS) by simultaneously inducing ubiquitin E3 ligase expression *via* feedback inhibition of PKB/Akt and proteasome biogenesis *via* Nuclear Factor Erythroid 2-Like 1 (Nrf1). Muscle growth was restored by reactivation of PKB/Akt, but not by Nrf1 knockdown, implicating ubiquitination as the limiting step. However, both PKB/Akt activation and proteasome depletion by Nrf1 knockdown led to an immediate disruption of proteome integrity with rapid accumulation of damaged material. These data highlight the physiological importance of mTORC1-mediated PKB/Akt inhibition and point to juxtaposed roles of the UPS in atrophy and proteome integrity.

## Abstract (web summary)

Exploring the relationship between mTORC1 and the ubiquitin-proteasome system, light is shed on the paradox between mTORC1-mediated muscle hypertrophy induced by PKB/Akt and the muscle atrophy induced by mTORC1 alone.

## Introduction

The protein kinase B (PKB)/Akt- mammalian target of rapamycin complex 1 (mTORC1)- Forkhead box O (FoxO) pathway is a central upstream signaling axis controlling skeletal muscle protein homeostasis (proteostasis), regulating both protein synthesis and breakdown, the latter via the autophagy/lysosomal system and the ubiquitin proteasome system (UPS). Activating PKB/Akt potently stimulates mTORC1-dependent protein synthesis and phosphorylates and sequesters FoxO transcription factors in the cytoplasm preventing their nuclear translocation and transcriptional activation of the UPS, thereby driving rapid skeletal muscle growth^1^. However, prolonged mTORC1 activity also promotes feedback inhibition of PKB/Akt via Ribosomal Protein S6 Kinase B1 (S6K1) and the Insulin Receptor Substrate 1 (IRS1)^2^. Thus, constant activation of mTORC1 by genetic elimination of the Tuberous Sclerosis complex subunit 1 (TSC1), an essential component of the mTORC1-inhibiting TSC complex, paradoxically not only fails to stimulate hypertrophy but induces atrophy in skeletal muscle, despite autophagy inhibition and the promotion of protein synthesis^3,4^. These observations implicate a parallel, mTORC1-mediated activation of protein breakdown pathways. Indeed, we have previously observed elevated levels of the key, atrophy-promoting E3 ubiquitin ligase *Trim63* (Muscle RING finger 1/MuRF1) and the E3 ubiquitin ligase member *Fbxo32* (atrogin-1/MAFbx) in muscle from mice with a muscle-specific knockout of the mTORC1 inhibitor *Tsc1* (TSCmKO mice)^4^, suggesting UPS activation. While strong activation of the UPS is considered the primary driver of muscle loss in many muscle-wasting conditions, the UPS is also the major system responsible for removing damaged proteins and can partially compensate for autophagy impairment to protect cellular homeostasis^5^. mTORC1 activation, autophagy inhibition, and UPS induction are common features of numerous muscle-wasting conditions including denervation and sarcopenia^4,6^. We have previously shown that high mTORC1 activity is a hallmark of sarcopenia, and that the TSCmKO mouse represents a model of premature muscle aging^6^. Therefore, we used TSCmKO mice as a model to understand the underlying mechanisms responsible for mTORC1-mediated UPS induction and its effect on muscle size and proteostasis.

The UPS is a highly sophisticated proteolytic system that involves targeted labeling of substrates with ubiquitin via sequential transfer from the E1 ubiquitin-activating enzyme to E2 ubiquitin-conjugating enzymes and then to one of many E3 ubiquitin ligases, which confer both substrate and ubiquitin chain conjugation specificity. For example, targets of the E3 ligase MuRF1 are thought to include major muscle structural proteins (e.g., titin, myosin heavy chains and some myosin light chains^7^), while atrogin-1/MAFbx is thought to target key proteins involved in protein synthesis (e.g., eIF3f ^8^). Substrates labeled with a chain of four or more K48-linked ubiquitin proteins are preferentially targeted to the functional unit responsible for UPS-induced protein degradation, which is referred to as the 26S proteasome based on its sedimentation coefficient. The 26S proteasome consists of the 20S catalytic core particle and one or two 19S regulatory particles. The 20S core particle is a cylindrical structure with a central pore made up of four stacked rings each containing seven individual proteasome subunit proteins. The inner two catalytic rings each contain PSMB1-7, while the outer two rings contain PSMA1-7 and act as a gate for substrate entry. The β5 (PSMB5), β1 (PSMB6), and β2 (PSMB7) catalytic enzymes are responsible for chymotrypsin-, caspase- and trypsin-like activity during protein cleavage, respectively. As the catalytic sites are located on the interior surface of the central pore, their activity relies on substrate access, a process mediated by 19S regulatory particles, which are responsible for recognizing and capturing (PSMD4, ADRM1), de-ubiquitinating (PSMD14) and unfolding ubiquitinated substrates before gate opening (PSMC1, 3 and 4) and substrate transport (PSMC1-6) via ATPase activity into the 20S core particle for destruction^9^.

Here, we found by transcriptomic and proteomic analyses that muscles from TSCmKO mice increase expression of many atrophy-related genes (aka “atrogenes”)^10,11^, including many E3 ubiquitin ligases, as well as most subunits of the 26S proteasome, resulting in increased proteasome activity. Inducible deletion of *Tsc1* in adult skeletal muscle (iTSCmKO mice) for as little as 10 days was sufficient to reproduce atrogene expression, proteasome subunit expression and activity as well as muscle wasting in fast, but not slow-twitch muscles (e.g., *soleus*). By detailed examination of signaling pathways, genetic activation of PKB/Akt and knockdown experiments, we identified nuclear factor, erythroid-derived 2-like 1 (Nfe2l1, also known as Nrf1) as the transcription factor responsible for 26S proteasome expression, while the PKB/Akt-FoxO axis was responsible for the expression of many ubiquitin-, stress- and autophagy-related atrogenes and muscle atrophy. While twenty days of PKB/Akt activation potently stimulated muscle hypertrophy in TSCmKO mice, it also triggered a severe myopathy, including p62-positive aggregate accumulation, vacuoles and impaired muscle function. Likewise, proteasome depletion by Nrf1 knockdown led to p62 accumulation and induction of atrogene expression. Together, these data point to juxtaposed roles of the UPS in skeletal muscle proteostasis, on the one hand driving muscle atrophy, but on the other alleviating cellular stress by removing misfolded and damaged proteins.

## Results

### Sustained skeletal muscle mTORC1 activity upregulates the ubiquitin proteasome system

**Fig. 1:**
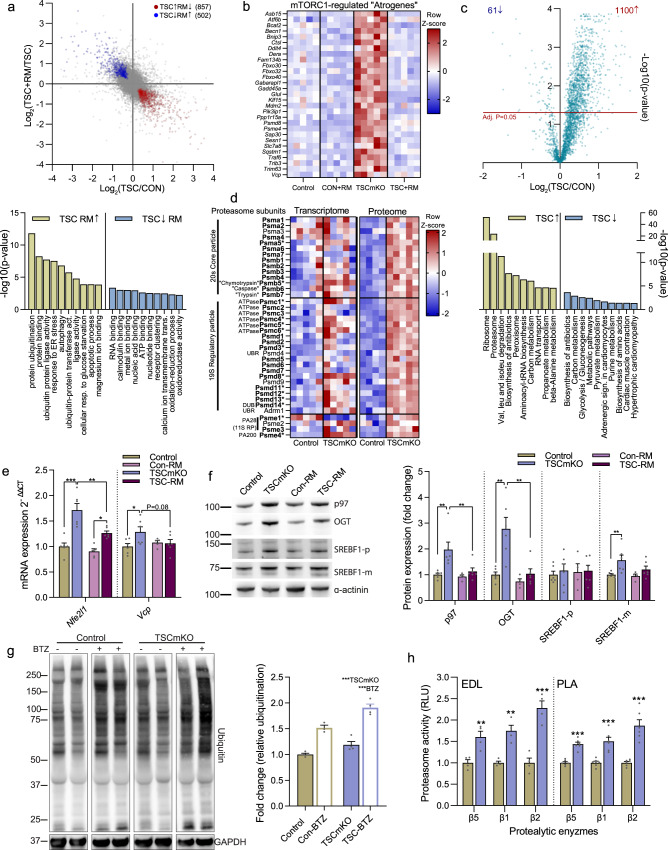
Skeletal muscle mTORC1 hyperactivity upregulates the ubiquitin proteasome system. **a** Pairwise comparison of muscle mTORC1 activation- (TSC/CON) and subsequent rapamycin inhibition-induced (TSC+RM/TSC) gene expression changes in EDL muscle with mTORC1-regulated genes up- (red) and downregulated (blue) in TSCmKO mice and counter-regulated by rapamycin as denoted. Top Gene Ontology (GO) terms are presented below (*n* = 5 per group). **b** Heatmap of changes in the expression of mTORC1-regulated “atrogenes”. **c** Volcano plot for changes in protein expression between TSCmKO and control TA muscle with top GO terms associated with significantly increased and decreased proteins below (*n* = 4–5 per group). **d** Heatmap of changes in gene and protein expression for control and TSCmKO mice of all expressed 26S proteasome subunits and members of the PA28 and PA200 proteasome activators. **e** mRNA and **f** protein expression of Nrf1 regulators, as measured by RT-qPCR (*gastrocnemius*) and Western blot (*tibialis anterior*), respectively (*n* = 4–6 per group). **g** Representative blots and quantification of mono- and poly-ubiquitinated proteins in control and TSCmKO mice treated with vehicle or 1 mg.kg^−1^ of the proteasome inhibitor Bortezomib (BTZ) 12–16 h before dissection (*n* = 3–4). **h** Luciferase-based peptidase activity of 20S proteasome catalytic enzymes in *extensor digitorum longus* (EDL; *n* = 4) and *plantaris* (PLA; *n* = 6) muscle. *Actb* was used as the reference gene (**e**) and either α-actinin (**f**) or GAPDH (**g**) was used as the protein loading control. Data are presented as mean ± SEM. Two-tailed Student’s *t*-tests (**h**) or two-way ANOVAs with Sidak post hoc tests were used to compare the data. *, **, and *** denote a significant difference between groups of *P* < 0.05, *P* < 0.01, and *P* < 0.001, respectively. For trends, where 0.05 < *P* < 0.10, *p* values are reported.

To understand the molecular mechanisms causing atrophy in muscles with high mTORC1 activation, we first analyzed the transcriptome of the fast-type *extensor digitorum longus* (EDL) muscle in 3-month-old TSCmKO mice^6^. A short-term (3 day) treatment with rapamycin was used to identify transcripts that responded acutely to mTORC1 inhibition. Differential expression (DE) analysis identified 857 and 502 transcripts with significantly (FDR < 0.05) higher and lower expression, respectively, in TSCmKO muscle compared to control that were also reversed by rapamycin (Fig. 1a, upper). According to DAVID^12^, the ‘biological process’ and ‘molecular function’ gene ontology (GO) terms that were most enriched in genes with higher expression in TSCmKO mice include multiple terms associated with the UPS (e.g., ‘protein ubiquitination’, ‘ubiquitin protein ligase activity’ and ‘ubiquitin-protein transferase activity’) as well as terms associated with ER stress and autophagy (Fig. 1a, lower). GO terms enriched in genes that are repressed by mTORC1 were related to binding of RNA, calmodulin, metal ions, nucleic acids, ATP, and nucleotides (Fig. 1a, lower). Next, we compared the 857 upregulated ‘mTORC1-regulated’ genes with a curated list of 249 atrophy-related genes, known from literature to be increased (154) or decreased (95) during experimental paradigms (e.g., starvation, diabetes, cachexia, denervation) of muscle atrophy (Supplementary data 1). Remarkably, 72 of the 154 increased atrophy-related genes were significantly (FDR < 0.05) upregulated in TSCmKO mice and almost half of them (29) were normalized (FDR < 0.05) by 3 days of rapamycin treatment (Fig. 1b). Many of the genes in this class are involved in ubiquitin-proteasome degradation, such as *Fbxo32* (atrogin1/MAFbx), *Trim63* (MuRF1), *Fbxo30* (MUSA), *Fbxo40*, *Mdm2*, *Traf6*, *Vcp (*p97*)*, *Psmd8 and Psme4* (PA200) or in autophagy-lysosomal degradation, such as *Becn1*, *Bnip3*, *Ctsl*, *Fam134b*, *Gabarapl1* and *Sqstm1* (p62)^10,13–19^.

To evaluate whether the protein degradation signature revealed by transcript analysis was also observed at the protein level, we used mass spectrometry (MS) to identify differentially expressed proteins from *tibialis anterior* (TA) muscle of 3-month-old TSCmKO mice and littermate controls. DE analysis identified 1101 significantly (*p* < 0.05) upregulated and 61 significantly downregulated proteins between control and TSCmKO mice (Fig. 1c, upper). The ribosome and proteasome were overwhelmingly the most prominently enriched KEGG pathways (Fig. 1c, lower). The ribosomal protein enrichment in TSCmKO mice is consistent with the central role of mTORC1 in the translation of ribosomal protein mRNA^20–22^. Of the 14 and ~21 proteins that make up the 20S core particle and the 19S regulatory particle of the 26S proteasome, 13 and 17 proteins, respectively, were significantly increased in TSCmKO muscle compared to control (Fig. 1d, bold), along with the proteasome activators Psme1, 3 and 4 (Fig. 1d). Although less prominent, many 26S proteasome subunits and activators were also significantly upregulated (denoted with an asterisk) at the transcript level (Fig. 1d). Confirming mRNA-seq data, RT-qPCR showed elevated expression of many atrogenes involved in ubiquitination, including the E3 ligases *Fbxo32, Trim63*, *Fbxo30*, *Mdm2* and *Traf6*, as well as the E3/E4 enzyme *Ube4b* and *Ubb* in TA muscle from 3-month-old TSCmKO mice compared to controls (Fig. S1a). Since the UPS is the main protein degradation pathway activated during muscle atrophy^23,24^, we examined the regulation of this pathway in further depth. Confirming mRNA-seq and proteomics data, RT-qPCR (Fig. S1a) and Western blot analysis (Fig. S1b) showed elevated gene and/or protein expression of numerous proteasome subunits in TSCmKO mice, including catalytic (*Psmb5*, *Psmb6* and *Psmb7)* and non-catalytic (*Psma1* and *Psma5)* 20S core particle subunits as well as ATPase (*Psmc1*) and non-ATPase (*Psmd8* and *Psmd11*) 19S regulatory particle components along with the proteasome activator *Psme4*.

mTORC1 activation is high in old, sarcopenic muscle, independent of PKB/Akt, and rapamycin or rapalogs attenuate sarcopenia^6,25,26^, suggesting that aging may cause a similar gene expression signature as seen in TSCmKO mice. To test this hypothesis, we analyzed atrogene and proteasome subunit gene expression in previously generated transcriptomic data (https://sarcoatlas.scicore.unibas.ch/) from *tibialis anterior* muscle of adult (10mCON), 30-month-old, sarcopenic (30mCON), and 30-month-old, rapamycin-treated mice (30mRM)^6^. Although changes were less strong in the aging data set, we saw a remarkably similar response to that seen in TSCmKO mice with approximately two thirds of mTORC1-regulated atrogenes (Fig. S2a) and over 80% of 26S proteasome subunits (Fig. S2b) being either higher (or tending to be higher) in 30mCON than 10mCON or lower (or tending to be lower) in 30mRM than 30mCON, or both. These data thus indicate that the age-induced activation of mTORC1 exerts similar responses as its genetic activation by depletion of TSC1.

The remarkably consistent and ubiquitous upregulation of proteasome subunits at both the transcript and protein levels suggests the involvement of a coordinating transcription factor. While FoxO transcription factors are known to control expression of many of the mTORC1-regulated ubiquitin- and autophagy-related atrophy genes, they are not known to broadly regulate the expression of proteasome subunits. Although currently uncharacterized in skeletal muscle, the nuclear factor E2-related factor 1 (Nfe2l1 or Nrf1) transcription factor has been identified as a master regulator of proteasome subunit gene expression, binding to the core antioxidant response element (ARE) sequence in the promoter region of all proteasome subunit genes^27–32^. Consistent with its involvement in the transcriptional regulation of the UPS in TSCmKO mice, mRNA expression of *Nfe2l1* and *Vcp*, which encodes the protein p97 and is responsible for Nrf1 transport from the ER lumen into the cytosol^17,33^, were significantly increased in TSCmKO muscles and reversed by 3 days of rapamycin treatment (Fig. 1e). Similarly, protein levels of the Nrf1 regulators p97, O-linked N-acetyl glucosamine (O-GlcNAc) transferase (OGT), which mediates O-GlcNAcylation and stabilization of Nrf1^34,35^, and the mature form of SREBF1, which mediates mTORC1-dependent induction of Nrf1^36,37^, were all significantly higher in TSCmKO than control mice (Fig. 1f). Three days of rapamycin treatment normalized protein levels of p97, OGT and the mature form of SREBF1 in TSCmKO mice, indicating that mTORC1 activity is responsible for the increase in Nrf1 regulators (Fig. 1f). In line with our observations, studies in cultured cells, brain and liver tissue have shown that genetic or physiological activation of mTORC1 increases *Nfe2l1* transcript levels and proteasome content through SREBF1^37^.

Despite the increase in proteasome content, ubiquitinated proteins were still higher in protein lysates from TSCmKO than control mice (Fig. 1g). Ubiquitinated protein accumulation was not the result of impaired proteasome degradation as both control and TSCmKO mice treated with the proteasome inhibitor Bortezomib displayed a similar increase in ubiquitinated proteins (Fig. 1g). Finally, a significant increase in 20S peptidase activity for all three proteolytic enzymes (i.e., chymotrypsin-like/β5, caspase-like/β1 and trypsin-like/β2) was seen in both EDL and *plantaris* (PLA) muscles of TSCmKO mice compared to controls (Fig. 1h). Altogether, these data show that sustained mTORC1 activity promotes an atrophy-like transcriptional program in skeletal muscle^11^, inducing the expression of atrophy-related E3 ligases as well as Nrf1 and a plethora of its 26S proteasome subunit targets, leading to increased proteasome activity.

### Acute TSC1 depletion rapidly activates the UPS in adult fast-type muscles

To further confirm that UPS induction in TSCmKO mice is a direct consequence of mTORC1 activation and not muscle adaptations to prolonged *Tsc1* deletion, we next examined mice in which recombination of the floxed *Tsc1* allele in skeletal muscle could be triggered by tamoxifen injection^38–40^. To assure successful recombination, mice also carried an EGFP-reporter only expressed after Cre recombinase-mediated removal of a stop cassette^38,41^. We analyzed control and inducible TSCmKO (iTSCmKO) mice 10 (10d-iTSCmKO) or 21 (21d-iTSCmKO) days after tamoxifen-induced recombination (Fig. S3a). Ten days after recombination, all *gastrocnemius* (GAS) muscle fibers were brightly GFP-positive, indicating successful recombination (Fig. S3b). Both, 10d- and 21d-iTSCmKO mice, displayed a strong reduction in TSC1 protein levels and a concomitant increase in phosphorylation status of the mTORC1 targets S6 (S240/S244 and S235/S236) and 4E-BP1 (S65), while the inhibitory feedback loop from S6K to PKB/Akt, indicated by reduced phosphorylation of PKB/Akt (S473) and PRAS40 (T246), was established in GAS muscle from 21d- but not 10d-iTSCmKO muscle (Fig. S3c). Similar results were observed in the slow-type *soleus* (SOL) muscle (Fig. S3d), which in contrast to fast-type muscle displays a progressive mass gain in TSCmKO mice^3,4^. Together, these data indicate rapid and efficient tamoxifen-inducible TSC1 depletion and mTORC1 activation in both fast- and slow-type muscles.

**Fig. 2:**
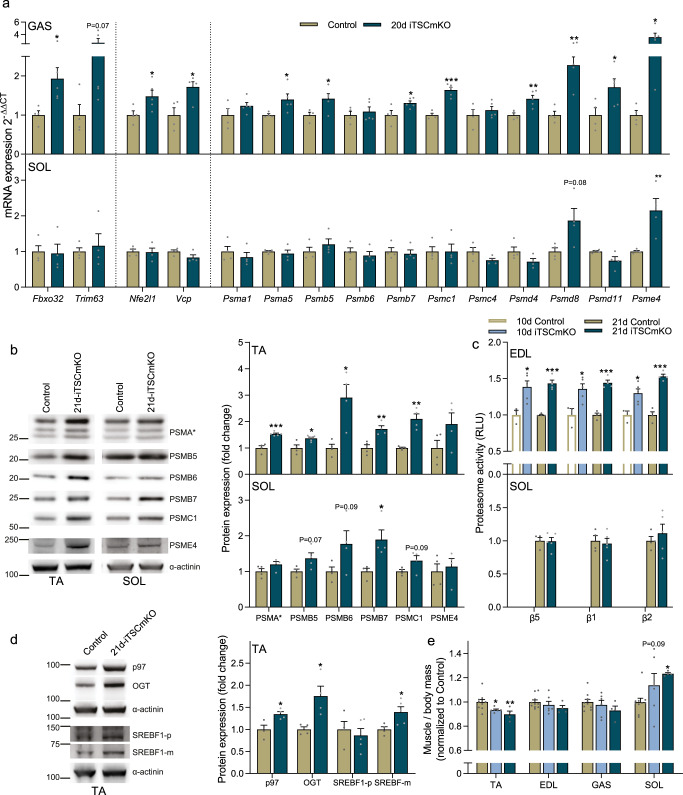
Acute TSC1 depletion rapidly activates the UPS in adult fast-twitch muscle. **a** RT-qPCR measures of mRNA expression for E3 ligases, Nrf1-related genes and selected proteasome subunits after 21 days of *Tsc1* deletion in *gastrocnemius* (GAS; upper panel) and *soleus* (SOL; lower panel). **b** Representative Western blots (left) and quantification (right) of 26S proteasome subunits and its activator in *tibialis anterior* (TA; upper) and SOL (lower). **c** Luciferase-based peptidase activity of 20S proteasome catalytic enzymes after 10 and 21 days recombination of floxed *Tsc1* alleles in *extensor digitorum longus* (EDL; upper) muscle and after 21 days recombination in SOL muscle (lower). **d** Representative Western blots (left) and quantification (right) of Nrf1 and its regulators in TA muscle. **e** muscle mass after 10 and 21 days recombination of floxed *Tsc1* alleles. Muscle mass of TA, EDL, GAS, and SOL was averaged across both limbs, normalized to body mass and then to control. *Tubb* or *Actb* were used as reference genes (**a**), while α-actinin was used as a protein loading control (**b**, **d**). For **a**, **b**, **d**, *n* = 4–5. For (**c**), *n* = 3–5. For **e**, *n* = 10 (Control), 6 (10d-TSCmKO) and 4 (21d-TSCmKO). Data are presented as mean ± SEM. Two-tailed Student’s *t*-tests (**a**–**d**) or one-way ANOVAs (**e**) with Fishers LSD post hoc tests were used to compare the data. *, **, and *** denote a significant difference between groups of *P* < 0.05, *P* < 0.01, and *P* < 0.001, respectively. For trends, where 0.05 < *P* < 0.10, *p* values are reported.

Supporting a rapid mTORC1-mediated UPS induction specifically in fast-type muscles, *Fbxo32* and *Trim63* (trend) were upregulated in fast-type GAS muscle from 21d-iTSCmKO mice (Fig. 2a, upper), along with a coordinated upregulation of *Nfe2l1*, *Vcp,* and 26S subunit genes. Aside from a moderate upregulation of *Psme4*, UPS-related gene expression was unaltered in SOL muscles from 21d-iTSCmKO compared to control mice (Fig. 2a, lower). Protein levels of key 26S proteasome subunits were also increased in GAS from 21d-iTSCmKO compared to control mice (Fig. 2b, upper) while this response was blunted in SOL muscle (Fig. 2b, lower). Furthermore, peptidase activity of the three catalytic β-proteasome subunits was strongly increased in fast-type EDL muscles of both 10d- and 21d-iTSCmKO mice compared to controls (Fig. 2c, upper), while activity was not altered after 21 days in SOL muscle (Fig. 2c, lower). In support of Nrf1 mediating proteasome biogenesis in TSCmKO mice, upregulation of the Nrf1 regulators p97, OGT and mature SREBF1 (Fig. 2d) mirrored proteasome subunit expression in fast-type 21d-iTSCmKO muscle. Importantly, UPS activation in fast-type, but not slow-type muscles of iTSCmKO mice correlated with progressive fast-type muscle mass loss and slow-type muscle mass gain, as previously observed in TSCmKO mice^3,4^, reaching statistical significance by 10 days in the TA and by 21 days in the SOL (Fig. 2e).

Together, these data demonstrate that mTORC1 rapidly activates the UPS, including atrophy-related ubiquitin E3 ligases and 26S proteasome biogenesis along with its transcriptional regulator Nrf1, in fast-type muscles just 21 days after floxed *Tsc1* allele recombination, which correlates with muscle atrophy. Hence, UPS activation is likely a direct result of mTORC1 activation rather than a consequence of the myopathic features associated with prolonged *Tsc1* deletion^4^. Interestingly, the regulation of UPS components in response to mTORC1 activation differs between fast- and slow-type muscles and provides a possible explanation for the differential muscle mass response. However, these data cannot distinguish between the possibility that this phenotype is based on a PKB/Akt-FoxO-regulated increase in atrophy-related gene expression or an Nrf1-induced upregulation of 26S proteasome content, or both. To answer this, we next examined the effect of proteasome biogenesis on skeletal muscle, reasoning that if the mTORC1-induced increase in proteasome activity overwhelms the mTORC1-induced increase in protein synthesis to drive muscle wasting, then perturbing proteasome biogenesis should shift proteostatic balance and increase muscle mass in TSCmKO mice.

### Nrf1 mediates the mTORC1-induced upregulation of the proteasome

**Fig. 3:**
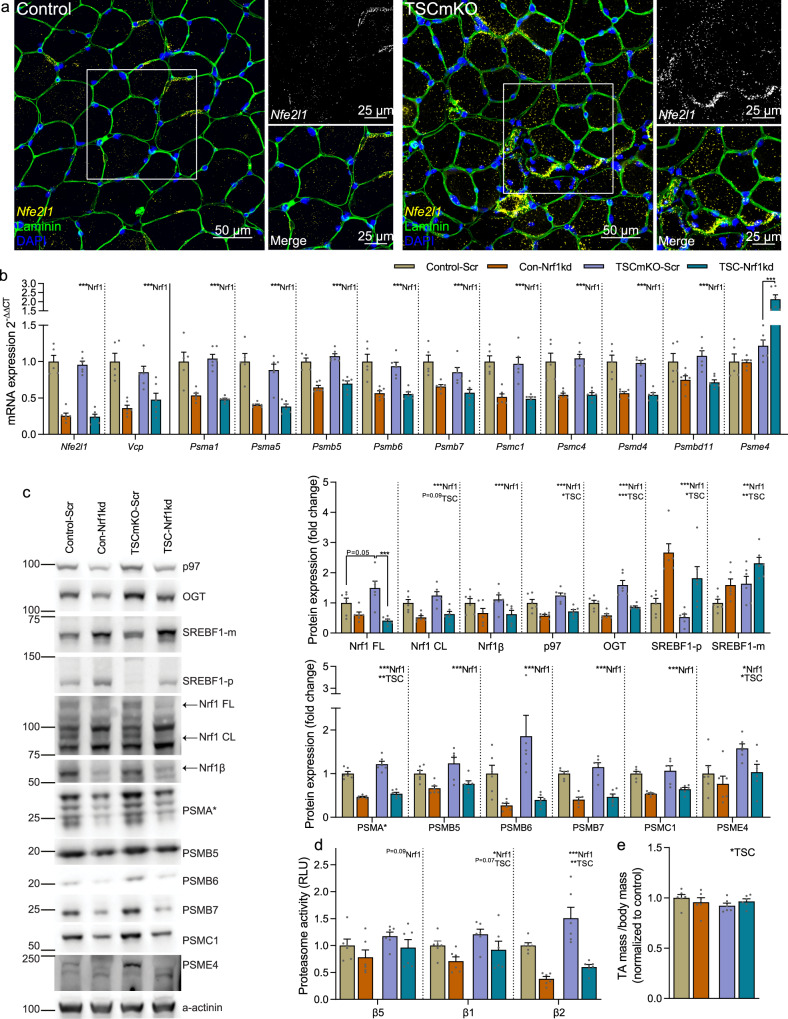
Nrf1 mediates mTORC1-stimulated proteasome biogenesis. **a** localization of *Nfe2l1* mRNA within muscle fibers using RNA in situ hybridization coupled to antibody-based immunofluorescence. **b** mRNA and **c** protein expression of Nrf1 (encoded by the *Nfe2l1* gene), Nrf1 regulators, 26S proteasome subunits and the 20S activator PSME4 in *tibialis anterior* (TA) muscle with representative Western blots (left). Nrf1 FL, CL, and β refer to the full length, cleaved and alternatively spliced versions of Nrf1, respectively. Muscles were collected 2 weeks after transfection of a plasmid expressing either a scrambled (Scr) or *Nfe2l1*-targeting (Nrf1-kd) shRNA into TA/*extensor digitorum longus* (EDL) muscles of control and TSCmKO mice. **d** Luciferase-based peptidase activity of 20S proteasome catalytic enzymes in EDL muscle and **e** TA muscle mass 2 weeks after transfection. *Des* was used as the reference gene and α-actinin as the protein loading control. For all experiments *n* = 6 per group, except for control in **b** where *n* = 5. Data are presented as mean ± SEM. Two-way ANOVAs with Sidak post hoc tests were used to compare the data. *, **, and *** denote a significant difference between groups of *P* < 0.05, *P* < 0.01, and *P* < 0.001, respectively. For trends, where 0.05 < *P* < 0.10, *p* values are reported.

To test how proteasome content affects muscle size, we targeted *Nfe2l1* with small hairpin RNA (shRNA). As muscle contains many non-muscle fiber cells that could also contribute to the increased expression of *Nfe2l1* in whole-muscle lysates, we first used single-molecule fluorescent in situ hybridization (smFISH; RNAscope®) to localize *Nfe2l1* expression. Indeed, the majority of *Nfe2l1* transcripts were expressed within skeletal muscle fibers in control mice and TSCmKO mice showed a strong increase of *Nfe2l1* puncta in the cytoplasm of muscle fibers with sporadic, peripherally localized clusters (Fig. 3a). These data show that muscle fiber mTORC1 activation triggers the increase in *Nfe2l1* mRNA within muscle fibers and supports the notion that *Nfe2l1* expression is a direct consequence of mTORC1 activation.

To test the efficacy of shRNA constructs to knock-down *Nfe2l1*, we first tested them in murine embryonic fibroblasts (MEFs). Cells were treated with Bortezomib for 4 h, which triggers the bounce-back response and causes the cleaved form of Nrf1 (Nrf1 CL) to accumulate in the cytosol (Fig. S4a)^28,33^. The most effective of the three shRNAs tested efficiently knocked-down cleaved Nrf1 (Nrf1 CL) and, to a lesser extent, full-length Nrf1 (Nrf1 FL) protein (Fig. S4b).

Next, we electroporated TA muscle of control and TSCmKO mice with *Nfe2l1*-targeting (Nrf1-kd) shRNA or scramble shRNA (Scr) as a control. Two weeks post-electroporation, *Nfe2l1* transcripts were more than 60% lower and *Vcp*, along with transcripts coding for all measured 26S proteasome subunit genes, but not the proteasome activator *Psme4*, were significantly reduced by Nrf1-kd shRNA in both control and TSCmKO mice compared to Scr shRNA (Fig. 3b). Similarly, Nrf1-kd shRNA strongly reduced the protein levels of Nrf1 FL in TSCmKO mice and both the cleaved and alternatively spliced (LCR-F1 or Nrf1β) Nrf1 isoforms along with p97 and OGT in both control and TSCmKO mice compared to a Scr shRNA (Fig. 3c). On the other hand, Nrf1-kd shRNA significantly increased both the premature and mature forms of SREBF1 in control and TSCmKO mice, suggesting a possible negative feedback loop between Nrf1 and SREBF1. Nrf1-kd shRNA also significantly reduced 26S proteasome protein expression, but unlike at the mRNA level, Nrf1-kd shRNA also moderately reduced PSME4 expression (Fig. 3c). Furthermore, Nrf1-kd shRNA significantly reduced β1 and β2 and tended to reduce β5 catalytic activity of the 20S proteasome in both control and TSCmKO mice (Fig. 3d). However, despite strong reductions in proteasome subunits and activity, knocking down Nrf1 was insufficient to boost skeletal muscle mass in either control or TSCmKO mice (Fig. 3e), suggesting that the Nrf1-induced upregulation of 26S proteasome content and activity does not contribute directly to mTORC1-induced skeletal muscle atrophy. Together, these results suggest that Nrf1 coordinately regulates basal proteasome content in healthy skeletal muscle and augments proteasome content in response to sustained mTORC1 activation but is unlikely to be directly responsible for driving atrophy.

### Feedback inhibition of PKB/Akt signaling drives mTORC1-mediated muscle atrophy

As the Nrf1-induced increase in 26S proteasome content and activity does not seem to cause smaller muscles in TSCmKO mice, we next asked whether feedback inhibition of PKB/Akt and subsequent release of FoxO inhibition could be responsible for mTORC1-driven muscle atrophy^2^. Under atrophic conditions, many atrophy-related genes, including E3 ubiquitin ligases, such as *Fbxo32*, *Trim63*, *Fbxo30,* and *Fbxo31* as well as the ubiquitin-conjugating factor *Ube4b* and the proteasome activator *Psme4* are predominately controlled by FoxO transcription factors^42,43^. To test this hypothesis, we crossed TSCmKO mice with AKT-TG mice expressing an active, myristoylated form of PKBα/Akt1, fused to EGFP and ERT2 in skeletal muscle^40^. In AKT-TG mice, the PKBα/Akt1-fusion protein is immediately degraded without tamoxifen-induced stabilization via ERT2 binding^44,45^.

**Fig. 4:**
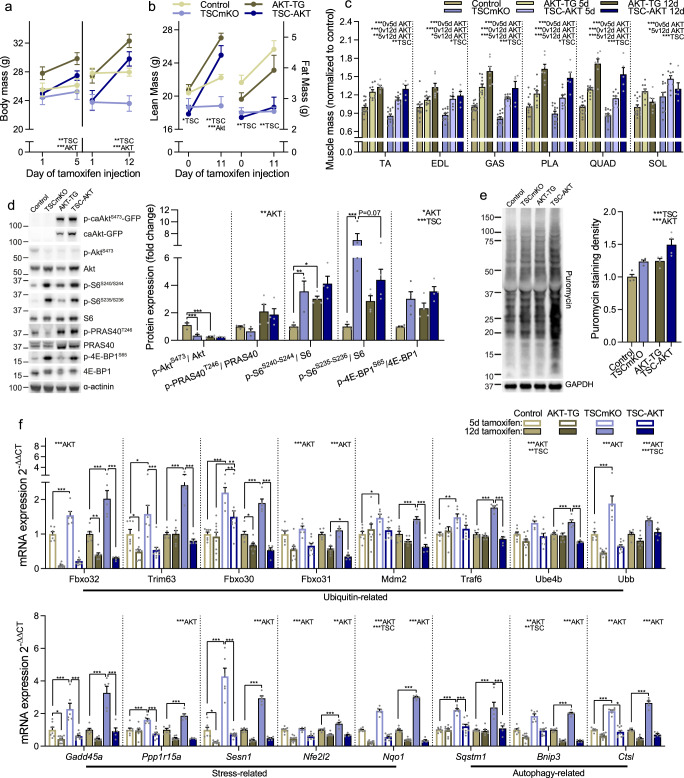
Feedback inhibition of PKB/Akt signaling drives mTORC1-mediated muscle atrophy by upregulating pro-atrophy, FoxO targets. **a** body mass before and after 5 (*n* = 7–10) or 12 (*n* = 5–7) days of tamoxifen treatment (40 mg kg^−1^ day^−1^) in control and TSCmKO mice and control (AKT-TG) and TSCmKO (TSC-AKT) mice expressing an active, myristoylated form of PKBα/Akt1, fused to EGFP and ERT2^40^. Changes in whole-body **b** lean (left) and fat (right) mass following 12 days of tamoxifen treatment (*n* = 5–7 per group) and **c** muscle mass after 5 and 12 days of tamoxifen treatment in control (*n* = 14), TSCmKO (*n* = 12), AKT-TG (*n* = 10 for 5d and 7 for 12d) and TSC-AKT (*n* = 9 for 5d and 5 for 12d) mice. **d** Western blots and quantification of phosphorylated and total proteins involved in the AKT-mTORC1 signaling pathway (*n* = 3–4; *tibialis anterior*) and **e** Western blots and quantification of newly synthesized proteins in muscles 30 min after puromycin injection in control, TSCmKO, AKT-TG and TSC-AKT mice treated with tamoxifen for 12 days (*n* = 4; *tibialis anterior*). **f** mRNA expression of ubiquitin- stress- and autophagy-related genes induced by sustained mTORC1 activity (*gastrocnemius*). For **f**, *n* = 7 (5d Con), 5 (5d TSCmKO), 9–10 (5d AKT-TG and TSC-AKT) and 5–7 (all 12d groups). *Tubb* was used as the reference gene for (**f**), while α-actinin (**d**) or GAPDH (**e**) were used as the protein loading control. Data are presented as mean ± SEM. Two way-ANOVAs with Sidak post hoc tests were used to compare data for each time point. *, **, and *** denote a significant difference between groups of *P* < 0.05, *P* < 0.01, and *P* < 0.001, respectively. For trends, where 0.05  <  *P*  <  0.10, *p* values are reported.

Control, AKT-TG, TSCmKO, and TSCmKO/AKT-TG (TSC-AKT) mice were injected daily with tamoxifen for 5 or 12 consecutive days. In AKT-TG and TSC-AKT mice, tamoxifen induced a rapid increase in body mass after both 5 and 12 days (Fig. 4a). Pre and post measures of body composition showed that lean mass accretion was primarily responsible for the increase in body mass after 12 days (Fig. 4b). Importantly, PKB/Akt activation potently reversed the fast-type muscle atrophy seen in TSCmKO mice, with fast-type muscle mass in both AKT-TG and TSC-AKT mice significantly heavier after 5 days of tamoxifen, and further increased after 12 days (Fig. 4c). Endogenous PKB/Akt activation was low in TSCmKO, AKT-TG, and TSC-AKT mice after 12 days of tamoxifen treatment. However, tamoxifen treatment strongly increased phosphorylation of the transgenic PKBα/Akt1-GFP fusion protein in AKT-TG and TSC-AKT mice, leading to higher phosphorylation of the PKB/Akt target PRAS40 (Fig. 4d). PKB/Akt activation increased phosphorylation of S6 at S240-244, but not significantly at S235-236, which was strongly phosphorylated in TSCmKO mice, in AKT-TG but not TSC-AKT mice. There was a main effect for PKB/Akt activation on 4E-BP1 (S65) phosphorylation, although the effect was also more pronounced in control mice (Fig. 4d). On the other hand, significant main effects were observed for both TSCmKO and PKB/Akt activation in protein synthesis, as determined by the incorporation of puromycin into newly synthesized proteins^46^ (Fig. 4e). Consistent with PKB/Akt activation suppressing FoxO, the expression of many ubiquitin- (*Fbxo32*, *Trim63*, *Fbxo30*, *Fbxo31*, *Mdm2*, *Traf6*, *Ube4b* and *Ubb*), stress- (*Gadd45a*, *Ppp1r15a*, *Sesn1, Nfe2l2* and *Nqo1*) and autophagy-related (*Sqstm1*, *Bnip3* and *Ctsl*) genes, the majority of which are known FoxO targets^43^, were suppressed in TSC-AKT compared to TSCmKO mice after 5 and/or 12d of tamoxifen treatment (Fig. 4f). In line with Nrf1 being primarily responsible for mTORC1-mediated proteasome biogenesis, PKB/Akt activation did not alter the majority of 26S proteasome subunits at the transcript (Fig. S5a) or protein level (Fig. S5b), with the notable exceptions of *Psmd8* and *Psme4* mRNA. However, despite having a limited impact on proteasome subunit content, prolonged PKB/Akt activation led to an increase in 20S peptidase activity (Fig. S4c). Together, these data indicate that the FoxO-mediated increase in atrophy-related genes, rather than the Nrf1-induced increase of 26S proteasome content, is responsible for shifting proteostatic balance towards muscle atrophy in TSCmKO mice.

### PKB/Akt reactivation compromises muscle integrity in TSCmKO mice

**Fig. 5:**
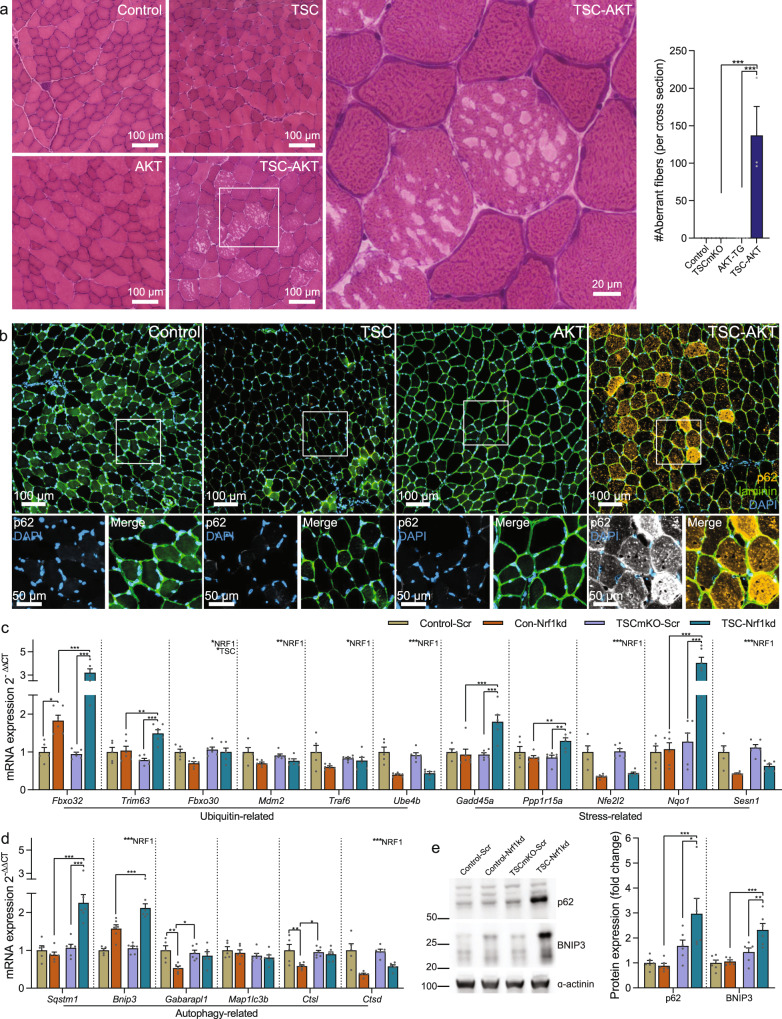
mTORC1-induced activation of the ubiquitin proteasome system preserves muscle integrity. **a** Representative images of Hematoxylin and eosin-stained cross sections and quantification of aberrant fibers and **b** representative images of *tibialis anterior* cross sections stained for p62 (yellow), laminin (green) and DAPI (blue) in control, TSCmKO (TSC), AKT-TG (AKT) and TSC-AKT mice after 12 days of tamoxifen treatment. **c** mRNA expression of ubiquitin- stress- and **d** autophagy-related genes in *gastrocnemius* muscle and **e** Western blots and quantification of autophagy-related protein expression in *tibialis anterior* muscle of control and TSCmKO mice 2 weeks after transfection of a plasmid expressing either a scrambled (Scr) or *Nfe2l1*-targeting (Nrf1-kd) shRNA. *Des* was used as the reference gene and α-actinin as the protein loading control. For **a**, *n* = 4, except for TSC-AKT where *n* = 3. For **c**–**e**, *n* = 6 per group, except for control in **c**, **d**, where *n* = 5. Data are presented as mean ± SEM. Two-way ANOVAs with Sidak post hoc tests were used to compare the data. *, **, and *** denote a significant difference between groups of *P* < 0.05, *P* < 0.01, and *P* < 0.001, respectively. For trends, where 0.05 < *P* < 0.10, p values are reported.

Although restoration of PKB/Akt activity in TSCmKO mice blocks atrophy-related gene expression and potently induces muscle growth, while examining the muscle structure of 12d TSC-AKT mice, we observed a striking accumulation of aberrant muscle fibers containing multiple vacuole-like structures (Fig. 5a). Since PKB/Akt blocks UPS induction, we hypothesized that damaged proteins that would normally be degraded by the UPS may be directed towards the autophagy/lysosomal system. However, since autophagy is blocked by sustained mTORC1 activity, protein aggregates marked for breakdown by the autophagy receptor p62 would accumulate. Indeed, strong p62 staining was observed in TA muscle fibers from TSC-AKT, but not control, TSCmKO or AKT-TG mice (Fig. 5b). Many fibers strongly positive for p62 staining also contained unstained regions indicative of the vacuoles observed in H&E stains. Importantly, p62 accumulation and vacuolated fibers are not normally seen in young TSCmKO mice and are rather characteristic of the late-onset myopathy typically observed at the age of 9–12 months^4^. This indicates that the mTORC1-driven increase in the expression of atrogenes regulated by FoxO transcription factors is a protective response that can compensate, at least initially, for sustained autophagy inhibition. We next wondered whether the Nrf1-mediated increase in proteasome content driven by mTORC1 also plays a similar role. To this end, we looked for signs of disturbed proteostasis in muscle from Control-Scr, Con-Nrf1kd, TSCmKO-Scr, and TSC-Nrf1kd mice. While Nrf1 depletion significantly suppressed some ubiquitin- stress- and autophagy-related genes, in both control and TSCmKO mice, it also increased the expression of key ubiquitin-related (*Fbxo32* and *Trim63*), stress-related (*Gadd45a*, *Ppp1r15a,* and *Nqo1*) and autophagy-related (*Sqstm1* and *Bnip3*) genes specifically in TSCmKO muscle (Fig. 5c, d). Furthermore, a significant accumulation of BNIP3 and p62 protein was observed in TSC-Nrf1kd compared to TSCmKO-Scr muscle (Fig. 5e), indicating an impaired capacity to degrade damaged proteins. Together, these data point to seemingly conflicting roles of UPS induction in response to sustained mTORC1 activity, on the one hand promoting muscle atrophy, while on the other hand compensating for autophagy blockade and preserving muscle integrity.

**Fig. 6:**
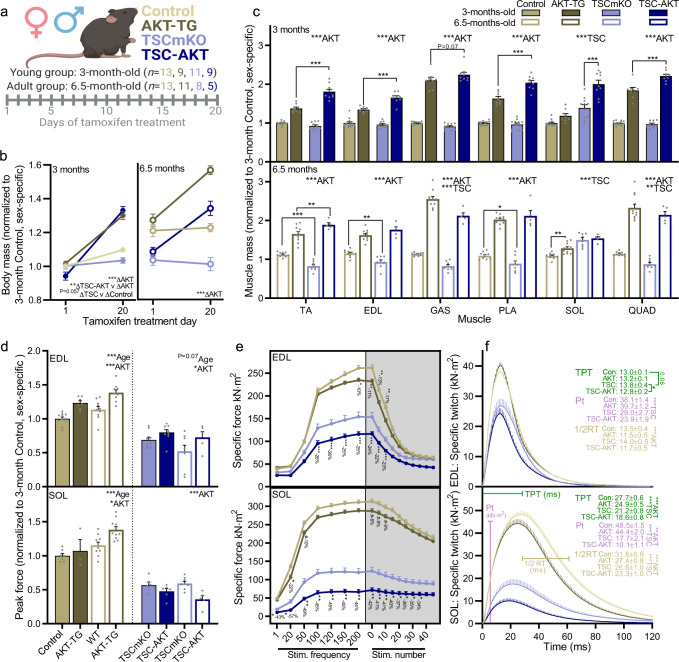
Dampened PKB/Akt activity preserves muscle function in young and adult TSCmKO mice. **a** Schematic of experimental design, including both young (3-month-old) and adult (6.5-month-old) groups. **b** Body mass before and after 20 days of tamoxifen treatment (40 mg kg^−1^ day^−1^) in 3-month-old (left, young) and 6.5-month-old (right, adult) control (*n* = 13 young and 10 adult), AKT-TG (*n* = 9 young and 11 adult), TSCmKO (*n* = 11 young and 8 adult) and TSC-AKT (*n* = 9 young and 5 adult) mice. **c** Muscle mass after 20 days tamoxifen treatment in young (upper) and adult (lower) mice. **d** Peak tetanic in vitro muscle force, normalized to 3-month-old control (sex specific), **e** specific force and **f** mean twitch profile for both fast-type EDL (upper) and slow-type SOL (lower) muscle. For **e**, **f**, *n* = 21, 17, 17 and 14 (EDL) and 16–17, 14, 14, and 11 (SOL) for control (Con), AKT-TG, TSCmKO, and TSC-AKT, respectively. Data are presented as mean ± SEM. Two-way ANOVAs with Sidak post hoc tests were used to compare data. For **e**, separate repeated measures two-way ANOVAs were used to compare between control and AKT-TG groups and between TSCmKO and TSC-AKT groups. *, **, and *** denote a significant difference between groups of *P* < 0.05, *P* < 0.01, and *P* < 0.001, respectively. For trends, where 0.05  <  *P*  <  0.10, *p* values are reported.

Next, we wanted to know how prolonged Akt activation in TSC-AKT mice would affect skeletal muscle integrity and function in both fast- (EDL) and slow-type (SOL) muscles. Furthermore, we wanted to know whether PKB/Akt-FoxO-mediated UPS activation also protected muscle integrity in older TSCmKO mice with a more progressed phenotype. To this end, we tamoxifen-treated 3- and 6.5-month-old control, TSCmKO, AKT-TG, and TSC-AKT mice for 20 consecutive days and performed ex vivo muscle function (Fig. 6a). Tamoxifen treatment strongly increased body (Fig. 6b) and fast-type muscle (Fig. 6c) mass in AKT-TG and TSC-AKT mice from both 3-month- and 6.5-month-old experimental groups. In contrast, PKB/Akt did not at all or only mildly induced muscle mass increases in the slow-type SOL muscle.

In line with changes in muscle mass, peak tetanic muscle force was markedly higher in EDL (Fig. 6d, upper) and slightly higher in SOL (Fig. 6d, lower) muscles from 3-month and 6.5-month-old AKT-TG compared to control mice. On the other hand, absolute peak tetanic muscle force was only mildly higher in EDL and significantly reduced in SOL muscles from TSC-AKT compared to TSCmKO mice for both 3- and 6.5-month-old mice. Since the functional response of muscle to AKT did not differ between 3- and 6.5-month-old mice, we pooled data for further analyses. Specific force at 200 Hz was slightly lower in EDL (~10%) and tended to be lower (~8%) in SOL muscle following PKB/Akt activation in control mice. In strong contrast, activation of PKB/Akt signaling in TSCmKO mice resulted in a 25% loss of specific force at 200 Hz in EDL (Fig. 6e, upper) and a 44% loss in SOL muscle (Fig. 6e). These results indicate a precipitous decline in muscle quality upon activation of PKB/Akt in TSCmKO mice. In line with the preferential fast-type muscle hypertrophy, 20 days of PKB/Akt activation resulted in faster twitch parameters with shorter half-relaxation times (1/2 RT) in both EDL (Fig. 6f, upper) and SOL (Fig. 6f, lower) muscles and a shorter time-to-peak tension (TPT) in SOL muscles in both AKT-TG and TSC-AKT compared to control and TSCmKO mice, respectively.

**Fig. 7:**
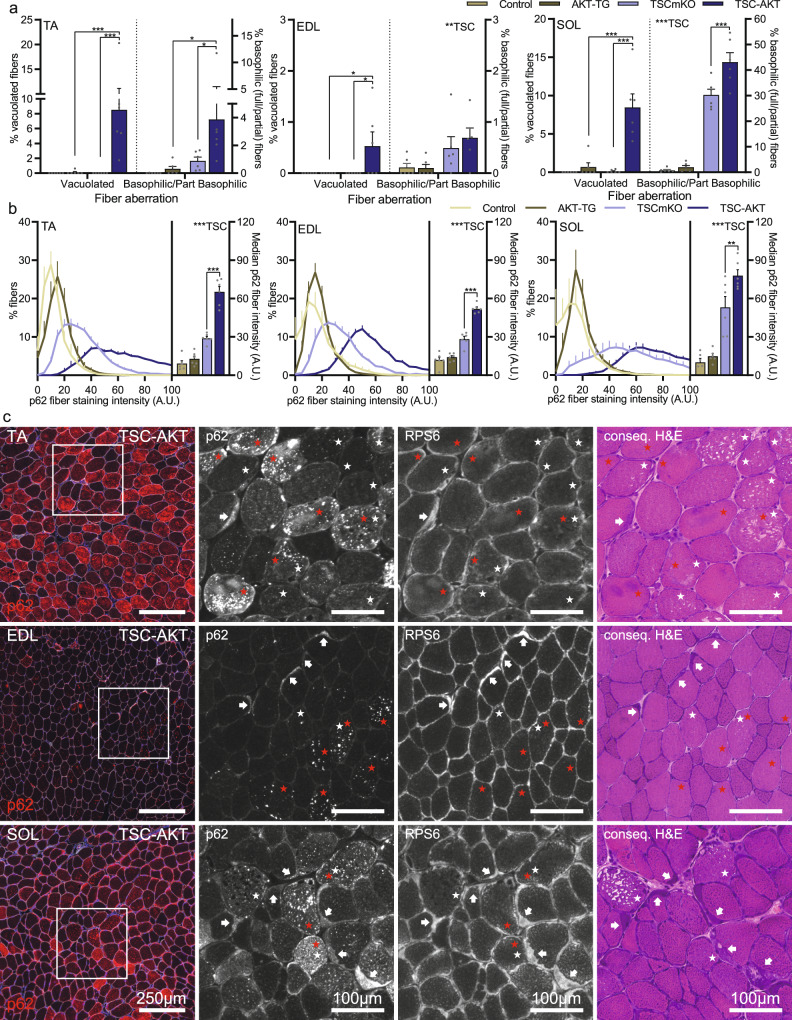
Fiber vacuolation and increased p62 staining occur in parallel, but as distinct phenotypes. **a** Quantification of aberrant fibers observed in Hematoxylin and eosin-stained cross sections from *tibialis anterior* (TA), *extensor digitorum longus* (EDL) and *soleus* (SOL) muscles collected from control, AKT-TG, TSCmKO and TSC-AKT mice. **b** Mean p62 fiber staining distribution (left) and median fiber staining intensity (right, column graph) for TA (left), EDL (middle) and SOL (right) muscles. **c** Representative images of *tibialis anterior* (TA), *extensor digitorum longus* (EDL) and *soleus* (SOL) cross sections showing p62 and laminin staining (left) and magnifications of p62 and RPS6 staining alongside consecutive H&E stained sections in TSC-AKT mice after 20 days of tamoxifen treatment. Vacuolated fibers (white star) and p62+ fibers (red star) frequently do not co-localize, while basophilic regions (arrows) invariably display high RPS6 staining and on occasions p62 staining. Data are presented as mean ± SEM, *n* = 6 per group. Two-way ANOVAs with Sidak post hoc tests were used to compare the data. *, **, and *** denote a significant difference between groups of *P* < 0.05, *P* < 0.01, and *P* < 0.001, respectively. For trends, where 0.05 < *P* < 0.10, *p* values are reported.

Loss in specific force usually reflects loss of “muscle quality”. We therefore examined the histology of cross-sections from three different muscles (EDL, SOL and TA) from all genotypes (Figs. 7a and S6). EDL muscles predominately express type IIB fibers, SOL muscles predominately express type I and IIA fibers, while TA displays an intermediate fast phenotype with a mix of IIB, IIX, and IIA fibers^6^. Vacuolated fibers were largely not visible in control, AKT-TG or TSCmKO mice across all three muscles. However, vacuolated fiber prevalence reached 8.5% in both the TA and SOL muscles, and, while occurring substantially less, EDL muscles from TSC-AKT mice also displayed significantly greater numbers of vacuolated fibers (0.5%) in TSC-AKT mice than either AKT-TG or TSCmKO mice (Fig. 7a). Fibers displaying basophilic regions, in some cases encompassing the entire fiber, were also more prevalent in all three examined muscles in TSC-AKT compared to TSCmKO or AKT-TG mice (Fig. 7a). Again, the prevalence of basophilic aberrations was substantially higher in SOL muscle, with more than 40% of TSC-AKT fibers displaying basophilic regions, compared to ~4% in TA and <1% in EDL muscle. In line with the muscle force data, and the protective effect of UPS activation in fast-type muscles of TSCmKO mice, basophilic aberrations, but not vacuoles, were also highly prevalent (30%) in SOL, but not TA or EDL muscles from TSCmKO mice.

**Fig. 8:**
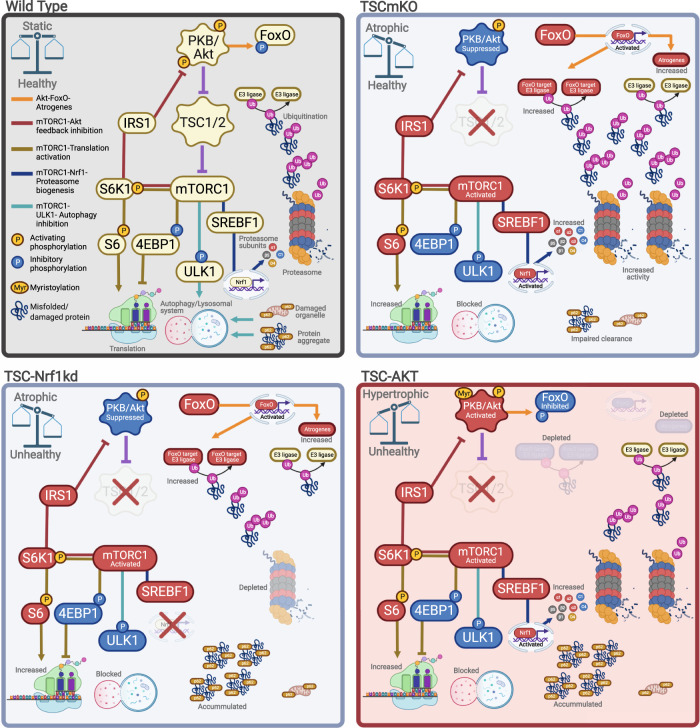
Proteostatic consequences of PKB/Akt-mTORC1 pathway manipulation in muscle. Major signaling networks involved in PKB/Akt-mTORC1-mediated muscle proteostasis in control mice (wild type, upper left), with sustained mTORC1 activity (TSCmKO, upper right), sustained mTORC1 activity and Nrf1-kd-mediated proteasome depletion (lower left) and sustained mTORC1 activity with constitutive PKB/Akt activation (lower right). Impairing ubiquitin-proteasome-mediated protein breakdown under conditions with high mTORC1 activity disrupts proteostasis and leads to unhealthy muscles, independent of muscle growth status. Figure created with BioRender.com.

Accumulation of p62 is a hallmark of the progressive myopathy seen in TSCmKO mice^4^ and this feature is exacerbated by activation of PKB/Akt (Fig. 5b). In an attempt to correlate accumulation of p62 with the pathological changes in the different muscles, we next quantified p62 fiber staining in TA, EDL and SOL muscles of the different genotypes. As shown after 12 days of PKB/Akt activation, TSC-AKT mice showed the highest p62 accumulation among genotypes after 20d tamoxifen treatment (Figs. 7b and S7). In line with the pathology severity between different muscles (Fig. 7a; SOL > TA > EDL), SOL contained the most fibers with high p62 staining while TA showed intermediate numbers and EDL the lowest p62 staining (Figs. 7b and S7). Finally, we also attempted to directly correlate pathological changes in individual muscle fibers from 20d TSC-AKT mice with molecular alterations. To this end, we stained cross-sections for p62 and for the ribosomal protein S6 and a consecutive section by H&E (Fig. 7c). However, while some vacuolated fibers (white stars) displayed high p62 staining (red stars) and vice versa, many non-vacuolated fibers displayed high p62 staining and many vacuolated fibers did not show high p62 staining, indicating that although these markers are co-regulated at the whole-muscle level, they represent different aspects of impaired protein homeostasis. In contrast, basophilic regions reliably overlapped with higher RPS6 staining (Fig. 7c, arrows), indicating ribosome accumulation. Together, these data indicate that the mTORC1-driven UPS activation mediated by PKB/Akt suppression is a protective response that compensates for sustained autophagy inhibition, at least for several months and the absence of this activation in slow-type muscles or suppression via PKB/Akt activation in all muscles is detrimental to proteostasis and muscle functionality (Fig. 8).

## Discussion

Skeletal muscle is highly dynamic, adapting its size to meet the specific needs of the body, with increased loading driving hypertrophy and disuse causing atrophy. An interconnected network of processes and signaling pathways act together to regulate changes in muscle size while maintaining the health of the proteome. There is strong evidence that the PKB/Akt-mTORC1-FoxO pathway lies at the heart of this network^47–50^, which led to the model that muscle size is determined by the balance between protein synthesis and degradation. According to this model, muscle mTORC1 activation, which increases protein synthesis and inhibits autophagy, should drive hypertrophy and block atrophy. However, this assumption does not hold true, with neither short- (Fig. 2) nor long-term^51^ mTORC1 activation, *via* muscle fiber-specific *Tsc1* deletion, resulting in fast-type muscle hypertrophy. On the contrary, chronic mTORC1 activation reduces fast-twitch fiber size and the mass of all fast-type muscles tested. Intriguingly and in opposite to fast-type muscles, the slow-type *soleus* muscle becomes hypertrophic in TSCmKO mice (Figs. 2, 4, 6) as well as in muscle-specific DEPDC5 KO mice, which also have chronically elevated muscle fiber mTORC1 activity^52^. However, despite promoting slow-type muscle hypertrophy, chronic mTORC1 activity consistently fails to increase muscle strength^4,52^, which is strongly impaired in both fast and slow-type muscles from TSCmKO mice (Fig. 6). Moreover, TSCmKO mice show many features consistent with accelerated muscle aging^6,53^. While the sarcopenia-like myopathy takes ~9 months to develop in TSCmKO mice^4,6^, with multiple lines of evidence implicating impaired autophagy as the mediating factor, mTORC1-driven muscle atrophy is already seen in 3-month-old TSCmKO mice^51^ and after just 21 days of tamoxifen-induced *Tsc1* deletion (Fig. 2). Here, we investigated the mechanisms underlying mTORC1-driven muscle atrophy in young, pre-myopathic TSCmKO mice and following acute TSC1 depletion in young mice, thereby avoiding influence from secondary consequences of the late-onset myopathy. Using genetic tools to activate and uncouple mTORC1 and PKB/Akt from potential upstream influences, shRNA-mediated proteasome depletion, and transcriptomic and proteomic profiling, we discovered that an mTORC1-driven upregulation of the ubiquitin proteasome system, including Nrf1-mediated proteasome biogenesis, outstrips increased translation and explains the counterintuitive atrophy in TSCmKO mice. Furthermore, we uncovered seemingly antagonistic roles of mTORC1-driven UPS activation in regulating muscle proteostasis, promoting atrophy while simultaneously maintaining muscle integrity.

UPS activation is a common feature of many muscle wasting conditions and loss and gain of specific E3 ligase function, such as atrogin-1^54^ blunts and promotes muscle atrophy, respectively. As such, perturbing UPS activity has received considerable attention as a potential means of tackling various muscle wasting conditions. Here we provide substantial evidence that hyperactive mTORC1 activates the UPS, including the induction of many FoxO-regulated, pro-atrophy E3 ligases through PKB/Akt suppression and ubiquitous upregulation of proteasome subunits *via* Nrf1, in fast but not slow-type muscles, accounting for their atrophic and hypertrophic responses to mTORC1 activation, respectively. Importantly, a similar increase in atrogene and proteasome subunit gene expression was observed in muscle from naturally aged mice (Fig. S2). Muscle mTORC1 activity was high in these aged mice and prolonged rapamycin treatment improved muscle size and function while lowering atrogene and proteasome subunit gene expression^6^.

Nrf1 transcriptionally promotes proteasome biogenesis by binding ARE sequences within the promoter region of all proteasome subunit genes, and its induction has been described, predominately in cultured cells, in response to proteasome inhibition^55^ and mTORC1 activity through SREBF1^36^. Here, we uncover a role for Nrf1-mediated proteasome biogenesis in response to sustained mTORC1 activity in skeletal muscle. While co-activation of UPS-mediated breakdown alongside protein synthesis by mTORC1 may seem like an exercise in futility, our data suggest this in fact represents a prudent strategy to cope with the inherent errors associated with protein translation, processing, and folding^56^. That is, mTORC1 may stimulate proteasome biogenesis to reduce the risk of proteotoxicity resulting from a build-up of damaged and misfolded proteins created by higher rates of protein synthesis. In support of this notion, UPS impairment either through PKB/Akt activation or proteasome depletion resulted in signs of proteotoxicity in TSCmKO mice. Misfolded proteins are predominately tagged with K48-linked ubiquitin chains and therefore preferentially degraded by the UPS. However, if the UPS is overwhelmed, ubiquitinated misfolded proteins can aggregate and form condensates through p62-mediated phase separation^57^. Since free mono-ubiquitin released by proteasomes during the de-ubiquitination step impairs p62-mediated phase separation, condensate formation is blocked when proteasome activity is appropriate^57^. If UPS activity is insufficient, p62-positive condensates would normally form and then be degraded through selective autophagy. We have previously observed widespread p62-positive condensate staining and vacuoles in muscle from TSCmKO mice^4^. However, these features do not become prevalent until 6-9 months of age, suggesting that Nrf1-mediated upregulation of the UPS by mTORC1 is initially sufficient to prevent a buildup of damaged/misfolded proteins and subsequent p62-positive condensate formation. In line with this, rapid p62 accumulation was observed within two weeks of UPS abrogation by either Nrf1-kd-mediated proteasome depletion or PKB/Akt-FoxO-dependent suppression of UPS induction (Fig. 5). However, the UPS cannot completely replace autophagic breakdown as it is structurally limited to the breakdown of unfolded proteins that fit within the core particle^9^. The late-onset accumulation of p62-positive condensates and vacuoles in TSCmKO mice could thus result from the accumulation of damaged organelles and other substrates that cannot physically be degraded by the UPS.

Our data also provides physiological context for the feedback inhibition from S6K1 to IRS1 on PKB/Akt. While this negative feedback loop blunts muscle growth, our data now indicate that it also serves to relieve the proteostatic burden of PKB/Akt activity and maintain muscle integrity. Indeed, chronic PKB/Akt activation has also been shown to disturb muscle proteostasis, with six months of constitutively active PKB/Akt expression also promoting p62-positive aggregates and vacuolated fibers along with other myopathic features^58^. The large time difference needed to trigger a proteotoxic response in TSCmKO and wild-type mice likely stems from inherent differences in the experimental systems. For example, TSC-AKT mice possess a pre-existing TSC1 KO-based proteostatic imbalance prior to PKB/Akt activation. Hence, the further accentuation of this imbalance by AKT activation may induce an immediate proteotoxicity, while the buildup of proteotoxic stress may require more time in wild-type mice^58^. In addition, mTORC1 is constantly active in the TSCmKO mice (as TSC1 is depleted by genetic means), while activation of PKB/Akt (and hence mTORC1) requires the constant presence of tamoxifen. Hence, it is not necessarily the case that mTORC1 is fully active throughout 24 h in AKT-TG mice despite the daily injection of tamoxifen. Further supporting the beneficial role of the negative feedback loop acting on PKB/Akt, we have recently shown that prolonged calorie restriction, which dampens PKB/Akt-activating stimuli, strongly reduces the accumulation of p62 and improves muscle function in old TSCmKO mice^26^.

While knockdown of Nrf1 and depletion of proteasomes in skeletal muscle led to increased signs of damaged protein accumulation, including increases in p62 and BNIP3, as well as induction of ubiquitin- (*Fbxo32* and *Trim63*) and stress- (*Gadd45a*, *Ppp1r15a,* and *Nqo1*) related atrogenes, the majority of which are FoxO targets, in TSCmKO muscle, Nrf1-kd was insufficient to rescue mTORC1-induced muscle atrophy. Therefore, it would seem that increased proteasome content alone is not sufficient to initiate proteasome degradation and therefore muscle atrophy; rather, the presence of FoxO-regulated enzymes involved in ubiquitination appears to be the limiting factor controlling muscle atrophy. Consistently, inhibition of one ubiquitin system gene, such as *Fbxo32*, *Trim63*, *Fbxo30* or *Traf6* is sufficient to limit protein degradation and attenuate muscle loss in various atrophic conditions^13,54,59^. Similarly, muscle-specific deletion of all three FoxO isoforms expressed in muscle (FoxO1, 3 and 4) is sufficient to attenuate fasting and denervation-induced loss of muscle mass and force^43^. In line with these observations, PKB/Akt activation strongly suppressed ubiquitin-related FoxO target genes and potently stimulated muscle hypertrophy in both control and TSCmKO mice (Fig. 4).

In a perfect world, only misfolded, damaged and obsolete signaling proteins would be targeted for destruction by the proteasome, however, the fact that overexpression of specific E3 ligases such as atrogin-1^54^ is sufficient to drive muscle wasting in otherwise healthy muscle tissue means that functional proteins are also caught in the cross hairs of FoxO-mediated UPS activation. A similar phenomenon appears to occur as a result of mTORC1-driven UPS induction, since (1) PKB/Akt-activation drives rapid muscle hypertrophy in TSCmKO mice and (2) the slow-twitch *soleus* muscle, which fails to induce E3 ligase gene expression (e.g., *Fbxo32* and *Trim63*) despite PKB/Akt suppression, also displays strong hypertrophy in TSCmKO mice. However, it is important to note that the mass gained by the SOL muscle in TSCmKO mice is largely non-functional, since muscle force is severely perturbed, as in fast-type muscles^4^ (Fig. 6).

Together, these data show that mTORC1-induced activation of the UPS has dual roles in muscle proteostasis, contributing to muscle atrophy through PKB/Akt suppression and FoxO-mediated induction of atrogenes, but simultaneously preserving muscle integrity by degrading damaged and misfolded proteins (Fig. 8). Since mTORC1 activation and autophagy impairment are frequently observed alongside UPS activation in atrophic conditions such as sarcopenia and denervation^4,6^, our data indicate that great caution should be exercised with intervention strategies designed to blunt the UPS as a means of reducing muscle atrophy. Furthermore, the evaluation of such intervention strategies should include measures of both muscle size and proteome health.

## Materials and methods

### Animal care

All procedures were performed in accordance with Swiss regulations for animal experimentation, and approved by the veterinary commission of the Canton Basel-Stadt. TSCmKO mice and their genotyping were previously described^3^. Controls were littermates floxed for *Tsc1* but not expressing HSA-Cre recombinase. Rapamycin (8 mg/kg; LC Laboratories) was administered I.P. as previously described^3^ for three consecutive days. *Tsc1*-floxed mice expressing a tamoxifen-inducible HSA-Cre (iTSCmKO) and their genotyping were previously described^40^. Littermates floxed for *Tsc1* but not expressing Cre recombinase were used as controls. To induce ablation of *Tsc1* in Cre recombinase-expressing muscle fibers, tamoxifen (Sigma-Aldrich) diluted in corn oil (Sigma-Aldrich) was administered I.P. at a dose of 75 mg kg day^−1^ for five consecutive days. The day after the fifth injection was defined as day 1 and Tamoxifen was administered again on days 3 and 4 (Fig. S3a). Both iTSCmKO mice and their controls received tamoxifen. AKT-TG mice expressing a tamoxifen-inducible constitutively active form of PKB/Akt were obtained from Dr. David Glass at Novartis Institutes for BioMedical Research (NIBR, Cambridge, MA, USA). Generation and genotyping of AKT-TG mice were previously described^40^. TSC-AKT mice were obtained by crossing *Tsc1*-floxed mice expressing HSA-Cre with AKT-TG mice. Control mice for TSC-AKT and AKT-TG mice were littermates floxed for *Tsc1* but not expressing Cre recombinase or the PKB/Akt transgene. AKT-TG, TSC-AKT mice and their respective controls were administered tamoxifen (40 mg kg day^−1^) I.P. for 5, 12 or 20 days. Body composition measurements were performed using an EchoMRI-100 (EchoMRI Medical Systems). Tissue was collected within 6 h after final tamoxifen injection on the 5th, 12th or 20th treatment day. For protein synthesis experiments, puromycin (0.04 μmol g^−1^; Sigma P8833) was administered I.P. exactly 30 min before dissection. Experiments were performed on 3–4 month-old female and male mice, except for those using tamoxifen in Fig. 2 and 4 where only male mice were used. Age-matched mice of the same sex were used for each individual experiment. Mice were maintained in a licensed animal facility with a fixed 12 h light-dark cycle (23 °C) and were allowed free access to food and water.

### Cell culture

SV40 immortalized mouse embryonic fibroblasts (MEFs) were maintained in Glutamax Dulbecco’s modified Eagle’s medium (DMEM Glutamax and pyruvate, Gibco) supplemented with 10% fetal bovine serum (FBS, Gibco) and 1% penicillin-streptomycin (pen/strep, Gibco) on cell culture dishes in 37 °C incubator with 5% CO_2_. For the Nrf1-kd experiment, MEFs were transfected with plasmids encoding for Nrf1-kd shRNA (see below) using Lipofectamine 2000 (Invitrogen). Forty-eight hours after transfection cells were scraped and lysed in ice cold RIPA buffer (50 mM TrisHCl pH 8.0, 150 mM NaCl, 1% NP-40, 0.5% sodium deoxycholate, 0.1% SDS, ddH_2_O) supplemented with phosphatase and protease inhibitors (Roche). DNA was sheared with a syringe (26G needle). Afterwards, the lysate was centrifuged at 16,000 × *g* for 20 min at 4 °C. Supernatant (cleared lysates) were used to determine total protein amount using the Pierce BCA Protein Assay Kit (Thermo Fisher Scientific) according to manufacturer’s protocol. For Nrf1 accumulation experiment, MEFs were treated with bortezomib (BTZ; 10 nM, Selleck Chemicals) or vehicle (DMSO; Sigma-Aldrich) for four hours prior to lysis.

### RNA extraction for RNA-seq

EDL muscles isolated from control and TSCmKO mice, were used to generate the corresponding cDNA-libraries. Poly-A mRNA was directly isolated from frozen EDL samples by using the Dynabeads^TM^ mRNA DIRECT^TM^ Purification Kit (61011, Invitrogen) with the Mini configuration followed by alkaline hydrolysis. To provide 5’ to 3’ directionality to fragmented samples, mRNA was treated with phosphatase and then with polynucleotide kinase (PNK). Further, 3’ and 5’ adaptors (Illumina) were ligated and the resulting product was reverse transcribed to generate cDNA by PCR. During PCR amplification, each prepared sample was uniquely indexed (barcoding) using index primer (Illumina) for multiplexing (differently indexed samples in one lane). PCR products have been purified twice with AMPure beads (Agencourt). The quality of the cDNA library was verified and quantified with a Bioanalyzer (Agilent Technologies). Illumina “HiSeq 2000” was used for sequencing at the Quantitative Genomics Facility (QGF) at the Department of Biosystems Science and Engineering (D-BSSE) of the ETH Zürich in Basel.

### Sample preparation LC-MS analysis

Approximately 5 μg of *gastrocnemius* muscle tissue was collected and lysed in 200 μl lysis buffer (2% sodium deoxycholate (SDC), 0.1 M ammoniumbicarbonate) using strong ultra-sonication (two cycles of sonication S3 for 10 seconds, Hielscher Ultrasonicator). Protein concentration was determined by BCA assay (Thermo Fisher Scientific) using a small sample aliquot. 50 μg of proteins were digested, reduced with 5 mM TCEP for 15 min at 95 °C and alkylated with 10 mM iodoacetamide for 30 min in the dark at 25 °C^60^. After diluting samples with 100 mM ammonium bicarbonate buffer to a final DOC concentration of 1%, proteins were digested by incubation with sequencing-grade modified trypsin (1/50, w/w; Promega, Madison, Wisconsin) overnight at 37 °C. Then, the samples were acidified with 2 M HCl to a final concentration of 50 mM, incubated for 15 min at 37 °C and the precipitated detergent removed by centrifugation at 10,000 g for 15 min. Subsequently, peptides were desalted on C18 reversed-phase spin columns according to the manufacturer’s instructions (Microspin, Harvard Apparatus) and dried under vacuum.

### TMT labeling and HpH-fractionation

The dried peptide samples were subsequently labeled with isobaric tag (TMT 10-plex, Thermo Fisher Scientific) according to the manufacturer’s instructions. To control for ratio distortion during quantification, a peptide calibration mixture consisting of six digested standard proteins mixed in different amounts were added to each sample before TMT labeling as recently described^60^. After pooling the TMT labeled peptide samples, peptides were again desalted on C18 reversed-phase spin columns according to the manufacturer’s instructions (Macrospin, Harvard Apparatus) and dried under vacuum. TMT-labeled peptides were fractionated by high-pH reversed phase separation using a XBridge Peptide BEH C18 column (3,5 µm, 130 Å, 1 mm × 150 mm, Waters) on an Agilent 1260 Infinity HPLC system. Peptides were loaded on column in buffer A (ammonium formate (20 mM, pH 10) in water) and eluted using a two-step linear gradient starting from 2% to 10% in 5 min and then to 50% (v/v) buffer B (90% acetonitrile/10% ammonium formate (20 mM, pH 10) over 55 min at a flow rate of 42 µl/min. The elution of peptides was monitored with a UV detector (215 nm, 254 nm). A total of 36 fractions were collected, pooled into 12 fractions using a post-concatenation strategy as previously described^61^, dried under vacuum and subjected to LC-MS/MS analysis.

### LC-MS analysis

In line with the previous studies^60^, the μRPLC-MS system was set up as follows. Chromatographic separation of peptides was carried out using an EASY nano-LC 1000 system (Thermo Fisher Scientific), equipped with a heated RP-HPLC column (75 μm × 37 cm) packed in-house with 1.9 μm C18 resin (Reprosil-AQ Pur, Dr. Maisch). Aliquots of 1 μg total peptides were analyzed per LC-MS/MS run using a linear gradient ranging from 95% solvent A (0.15% formic acid, 2% acetonitrile) and 5% solvent B (98% acetonitrile, 2% water, 0.15% formic acid) to 30% solvent B over 90 min at a flow rate of 200 nl/min. Mass spectrometry analysis was performed on Q-Exactive HF mass spectrometer equipped with a nanoelectrospray ion source (both Thermo Fisher Scientific). Each MS1 scan was followed by high-collision-dissociation (HCD) of the 10 most abundant precursor ions with dynamic exclusion for 20 s. Total cycle time was ~1 s. For MS1, 3e6 ions were accumulated in the Orbitrap cell over a maximum time of 100 ms and scanned at a resolution of 120,000 FWHM (at 200 *m/z*). MS2 scans were acquired at a target setting of 1e5 ions, accumulation time of 100 ms and a resolution of 30,000 FWHM (at 200 *m/z*). Singly charged ions and ions with unassigned charge state were excluded from triggering MS2 events. The normalized collision energy was set to 35%, the mass isolation window was set to 1.1 *m/z* and one microscan was acquired for each spectrum.

### Database searching and protein quantification

The acquired raw-files were converted to the mascot generic file (mgf) format using the msconvert tool (part of ProteoWizard, version 3.0.4624 (2013-6-3)). Using the MASCOT algorithm (Matrix Science, Version 2.4.1), the mgf files were searched against a decoy database containing normal and reverse sequences of the predicted SwissProt entries of *mus musculus* (www.ebi.ac.uk, release date 2015/08/04), the six calibration mix proteins^60^ and commonly observed contaminants (in total 34,204 sequences) generated using the SequenceReverser tool from the MaxQuant software (Version 1.0.13.13). The precursor ion tolerance was set to 10 ppm and fragment ion tolerance was set to 0.02 Da. The search criteria were set as follows: full tryptic specificity was required (cleavage after lysine or arginine residues unless followed by proline), three missed cleavages were allowed, carbamidomethylation (C), TMT6plex (K and peptide n-terminus) were set as fixed modification and oxidation (M) as a variable modification. Next, the database search results were imported to the Scaffold Q + software (version 4.3.2, Proteome Software Inc., Portland, OR) and the protein false identification rate was set to 1% based on the number of decoy hits. Protein probabilities were assigned by the Protein Prophet program^62^. Proteins that contained similar peptides and could not be differentiated based on MS/MS analysis alone were grouped to satisfy the principles of parsimony. Proteins sharing significant peptide evidence were grouped into clusters. Acquired reporter ion intensities in the experiments were employed for automated quantification and statistical analysis using a modified version of our in-house developed SafeQuant R script (v2.3^60^). This analysis included adjustment of reporter ion intensities, global data normalization by equalizing the total reporter ion intensity across all channels, summation of reporter ion intensities per protein and channel, calculation of protein abundance ratios and testing for differential abundance using empirical Bayes moderated t-statistics. Finally, the calculated *p*-values were corrected for multiple testing using the Benjamini−Hochberg method.

### Data processing

Mass-spectrometric data were statistically validated by the SafeQuant software tool^63^ developed in house. Proteins were considered as significantly differentially expressed with an adjusted *p* value of 0.05. Characterization and enrichment analysis of the differentially expressed proteins was done using DAVID analysis and functional annotation clustering^12,64^.

### RT-qPCR

Dissected muscle was rapidly frozen in liquid nitrogen. Total RNA was extracted using the RNeasy Mini Kit (Qiagen) according to manufacturer’s protocol. Equal amounts of RNA were transcribed into cDNA using the iScript cDNA Synthesis Kit (BioRad). Selected genes were amplified and detected using the Power SYBR Green PCR Master Mix (Applied Biosystems) or FastStart Essential DNA Green Master (Roche). Quantitative expression was determined by StepOnePlus Real-Time PCR System (Applied Biosystems) or LightCycler 480 (Roche). Data were analyzed using the comparative Cq method (2 − ΔΔCq). Raw Cq values of target genes were normalized to Cq values of a housekeeping gene (*Tubb*, *Actb* or *Des*), which was stable between conditions, and then further normalized to the control group for ease of visualization. Primers used are outlined in Table S1.

### In vitro muscle force

In vitro muscle force was measured in EDL and SOL muscles carefully excised and mounted on the 1200 A Isolated Muscle System (Aurora Scientific, Aurora, ON, Canada) in an organ bath containing 60 mL of Ringer solution (137 mM NaCl, 24 mM NaHCO_3_, 11 mM glucose, 5 mM KCl, 2 mM CaCl_2_, 1 mM MgSO_4_, and 1 mM NaH_2_PO_4_) gassed with 95% O_2_, 5% CO_2_ at 30 °C. After defining the optimal length, muscles were stimulated with 15-V pulses. Muscle force was recorded in response to 500-ms pulses at 10–250 Hz. Muscle fatigue was assessed by 6 min of repeated tetanic stimulations at 200 Hz for EDL and 120 Hz for SOL, respectively, separated by 8 s.

### Western blot analysis

Dissected muscle was rapidly frozen in liquid nitrogen. Frozen muscles were pulverized on a metal plate chilled in liquid nitrogen and directly snap-frozen as a pellet in liquid nitrogen. Samples were lysed in ice cold RIPA buffer (50 mM TrisHCl pH 8.0, 150 mM NaCl, 1% NP-40, 0.5% sodium deoxycholate, 0.1% SDS, ddH_2_O) supplemented with phosphatase and protease inhibitors (Roche), incubated on a rotating wheel for 2 h at 4 °C and sonicated twice for 10 s. Afterwards, the lysate was centrifuged at 16,000 × *g* for 20 min at 4 °C. Supernatant (cleared lysates) were used to determine total protein amount using the Pierce BCA Protein Assay Kit (Thermo Fisher Scientific) according to manufacturer’s protocol. Proteins were separated on 4–12% Bis-Tris Protein Gels (NuPage Novex, Thermo Fisher Scientific) and transferred to nitrocellulose membrane (GE Healthcare Life Sciences, Amersham). The membrane was blocked with 5% BSA, 0.1% Tween-20, PBS for 1 h at room temperature. The primary antibody diluted in the blocking solution was incubated overnight at 4 °C with continuous shaking. The membranes were washed with PBS-T (0.1% Tween-20, PBS) for 7 min three times and incubated with secondary horseradish peroxidase-conjugated (HRP) antibody for 1 h at room temperature. After washing with PBS-T, proteins were visualized by chemiluminescence (KPL LumiGLO^Ⓡ^, Seracare). Signal was captured on a Fusion Fx machine (VilberLourmat) and analyzed with FUSION Capt FX software. All antibodies used for immunoblotting are listed in Table S2.

### Proteasome activity assay

Proteasome activity measurements were adapted from a protocol described previously (Strucksberg et al.^65^). Briefly, dissected *extensor digitorum longus* (EDL), *soleus* (SOL) or *plantaris* (PLA) muscles were rinsed in ice-cold PBS, immediately cut into 5–6 pieces and directly lysed in ice-cold PBS-E (5 mM EDTA pH 8.0, PBS pH 7.2) and sonicated two times for 10 s. Afterwards, the lysate was centrifuged at 13,000 × *g* for 5 min at 4 °C. Supernatant (cleared lysates) were used to determine total protein amount using the Pierce BCA Protein Assay Kit (Thermo Fisher Scientific) according to manufacturer’s protocol. Three individual luciferase-based Proteasome-Glo^TM^ Assay Systems (Promega) were used to measure the activity of each peptidase of the proteasome. Assay was performed on white, 96-well microplates (greiner BIO-ONE) and luminescence was measured with an Infinite M1000 (Tecan). Human, purified 20S proteasome (Enzo Life Sciences, BML-PW8720) was used as a positive control. Proteasome inhibitor MG-132 (50 µM, TOCRIS Biotechne) was used to subtract non-proteasomal background activity.

### Histology analysis

Cryostat sections (10 μm) were cut from TA, EDL, and SOL as previously described^6^. For histological analysis, sections were stained with hematoxylin and eosin (H&E; Merck, Zug, Switzerland). For p62 and RPS6 staining, sections were fixed in 4% PFA for 10 min, washed in PBS for 10 min and then blocked and permeabilized in PBS containing 10% goat serum and 0.4% triton X-100 for 1 h at RT before being incubated overnight at 4 °C in a primary antibody solution (10% goat serum in PBS) containing primary antibodies against p62/SQSTM1 (GP62-C, 1:200, Progene), RPS6 (5G10; 1:200, #2217, Cell Signaling) and Laminin-α2 (L0663, Sigma). Sections were then washed 4 × 10 min in PBS before being incubated in antibody solution containing the secondary antibodies Alexa488 (#112-545-008, 1:200, Jackson), Alexa647 (#711-605-152, 1:200, Jackson) and Cy3 (#706-165-148, 1:200, Jackson). Sections were washed 4 × 10 min in PBS, mounted with ProLong Gold Antifade (Invitrogen, P36941) and imaged at the Biozentrum Imaging Core Facility with an Axio Scan.Z1 Slide Scanner (Zeiss- Oberkochen, Germany) equipped with appropriate band-pass filters. Fiji macros were developed in-house to allow an automated analysis of muscle fibers based on cell segmentation and their staining intensity. All macros and scripts used in this study are available upon request.

### RNAscope

Slides were fixed in cold 4% PFA for 15 min at 4 °C before serial dehydration for 5 min in each of 50%, 70% and 2 × 100% ethanol. Slides were then dried for five min at RT and circled with a Hydrophobic Barrier Pen (Vector Laboratories, GZ-93951-68). Sections underwent protein digestion (protease IV) for 30 min and then 15 min at RT before being washed twice with PBS. RNA hybridization with probes against *Nfe2l1* (580611, Advanced Cell Diagnostics) and subsequent amplification steps were performed according to the manufacturer’s instructions at 40 °C in a HybEZ^TM^ oven (Advanced Cell Diagnostics). After hybridization, Slides were blocked 60 min at RT in PBS containing 0.4% Trition X-100 and 10% goat serum, washed 2 × 5 min in PBS and then incubated with primary antibodies against Laminin-α2 (#11576, Abcam) in antibody solution containing 10% goat serum in PBS. Slides were then washed 4 × 10 min in PBS and incubated with GARt-488 (112-545-008, Jackson) secondary antibody and DAPI in antibody solution. Slides were then washed 4 × 10 min in PBS and mounted with ProLong^TM^ Gold antifade (Invitrogen).

### In vivo muscle transfection and electroporation

The methods to construct plasmid vectors encoding shRNA have been described elsewhere^66^. The murine 19 nucleotide target sequences corresponding to: GGC CCG ATT GCT TCG AGA A (Nrf1) and pRFP-C-RS scrambled shRNA plasmid vectors were obtained from OriGene (TR30015). For analgesia, mice were administered Buprenorphine (0.1 mg/kg) subcutaneously before and every 4–6 h during the day and in drinking water overnight for 2 days and nights after electroporation. Mice were anesthetized with isoflurane. The *tibialis anterior* (TA) muscle was exposed and injected with 50 μl (8 IU) of hyaluronidase (Sigma) two hours before being injected with 50 μl of 2 μg/μl shRNA plasmid DNA. Electroporation was then performed by applying three 30 ms pulses of 150 V/cm with a 50 ms pulse interval using a NEPA21 electroporation system (NepaGene). Mice were analyzed 2 weeks after electroporation.

### In vitro muscle force

In vitro muscle force was measured in the fast-twitch *extensor digitorum longus* (EDL) and slow-twitch *soleus* muscles. After careful isolation, muscle tendons were tied with surgical suture at each end and mounted on the 1200 A Isolated Muscle System (Aurora Scientific, Aurora, ON, Canada) in an organ bath containing 60 mL of Ringer solution (137 mM NaCl, 24 mM NaHCO_3_, 11 mM Glucose, 5 mM KCl, 2 mM CaCl_2_, 1 mM MgSO_4_, 1 mM NaH_2_PO_4_) gassed with 95% O_2_; 5% CO_2_ at 30 °C. After defining optimal length, muscles were stimulated with 15 V pulses. Muscle force was recorded in response to 500 ms pulses of 10–250 Hz. Muscle fatigue was assessed by 6 min of repeated tetanic stimulations at 200 Hz for EDL and 120 Hz for SOL, respectively, separated by 8 s.

### Statistics and reproducibility

All values are expressed as mean ± SEM, unless otherwise stated. Data were tested for normality and homogeneity of variance using a Shapiro–Wilk and Levene’s test, respectively. Data were analyzed in GraphPad Prism 9. Student’s *t* tests were used for pairwise comparisons, while one-way ANOVAs with Fisher’s LSD post hoc tests were used to compare between three groups, so long as the ANOVA reached statistical significance. Two-way ANOVAs with Sidak post hoc tests, or two-way repeated-measure ANOVAs for multiple recordings over time, were used to compare between groups with two independent variables. Both significant differences (*P* < 0.05) and trends (*P* < 0.1) are reported where appropriate. For each model, multiple independent experiments were conducted either using different treatment times (iTSCmKO and TSC-AKT studies) or different sets of mice (TSC-RM and Nrf1-kd studies) with comparable results.

### Reporting summary

Further information on research design is available in the Nature Research Reporting Summary linked to this article.

## Supplementary information

Supplementary Material

Description of Additional Supplementary Files

Supplementary Data

Reporting summary

## Data Availability

RNA-seq data were previously reported^6^ and deposited at the Gene Expression Omnibus GEO)^67^ under accession number GSE139214. Additionally, the data are freely available using the web-based application, SarcoAtlas (https://sarcoatlas.scicore.unibas.ch/). The mass spectrometry proteomics data have been deposited to the ProteomeXchange Consortium via the PRIDE^68^ partner repository with the dataset identifier PXD034117 and 10.6019/PXD034117. Full uncropped Western blots are provided in Figs. S8-19. The source data for graphs is provided in the Supplementary Data.
