# Supplementary material for: Dual roles of mTORC1-dependent activation of the ubiquitin-proteasome system in muscle proteostasis
Source: Commun Biol. 2022 Oct 27;5:1141. doi: 10.1038/s42003-022-04097-y (PMC9613904; doi:10.1038/s42003-022-04097-y)
Supplement: Supplementary file 1 — Supplementary Material [file 42003_2022_4097_MOESM1_ESM.pdf]

SUPPLEMENTARY FIGURES

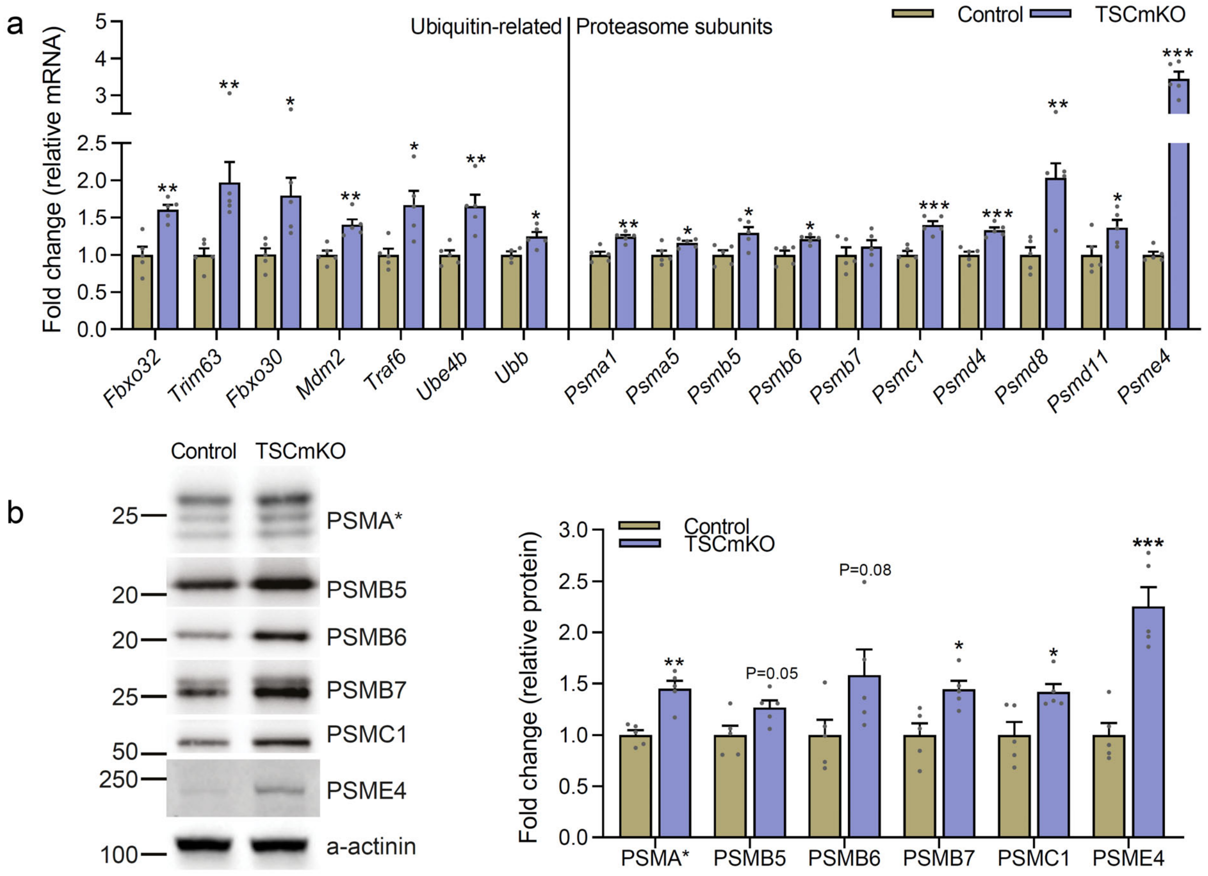

Figure S1. (a) mRNA expression of ubiquitin-related (left) and proteasome subunit (right) genes measured by RT-qPCR and **(b)** western blots and quantification of protein expression of 26S proteasome subunits and the 20S activator PSME4 in *gastrocnemius* (mRNA) and *tibialis anterior* (protein) muscle from control and TSCmKO mice. Data are presented as mean ± SEM. Two-tailed Student's t-tests were used to compare the data. *, **, and *** denote a significant difference between groups of $P < 0.05$, $P < 0.01$, and $P < 0.001$, respectively. For trends, where $0.05 < P < 0.10$, P values are reported.

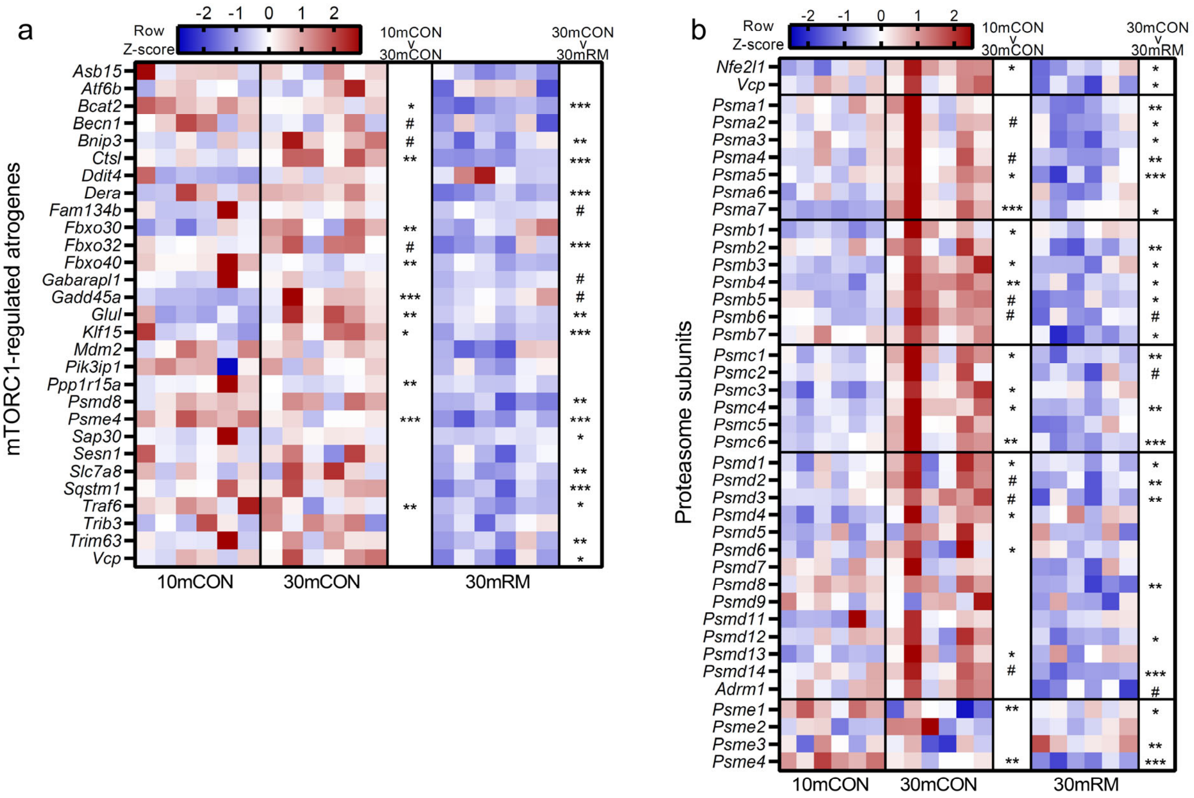

Figure S2. Heatmap of changes in mRNA expression of **(a)** mTORC1-regulated atrogenes and **(b)** 26S proteasome subunits and members of the PA28 and PA200 proteasome activators in wild-type adult mice (10mCON), 30-month-old, sarcopenic mice (30mCON) and 30-month-old mice treated from 15 months of age with rapamycin (30mRM) [6]. *, **, and *** denote a significant difference between groups of $P < 0.05$, $P < 0.01$, and $P < 0.001$, respectively. # denotes a trend, where $0.05 < P < 0.10$.

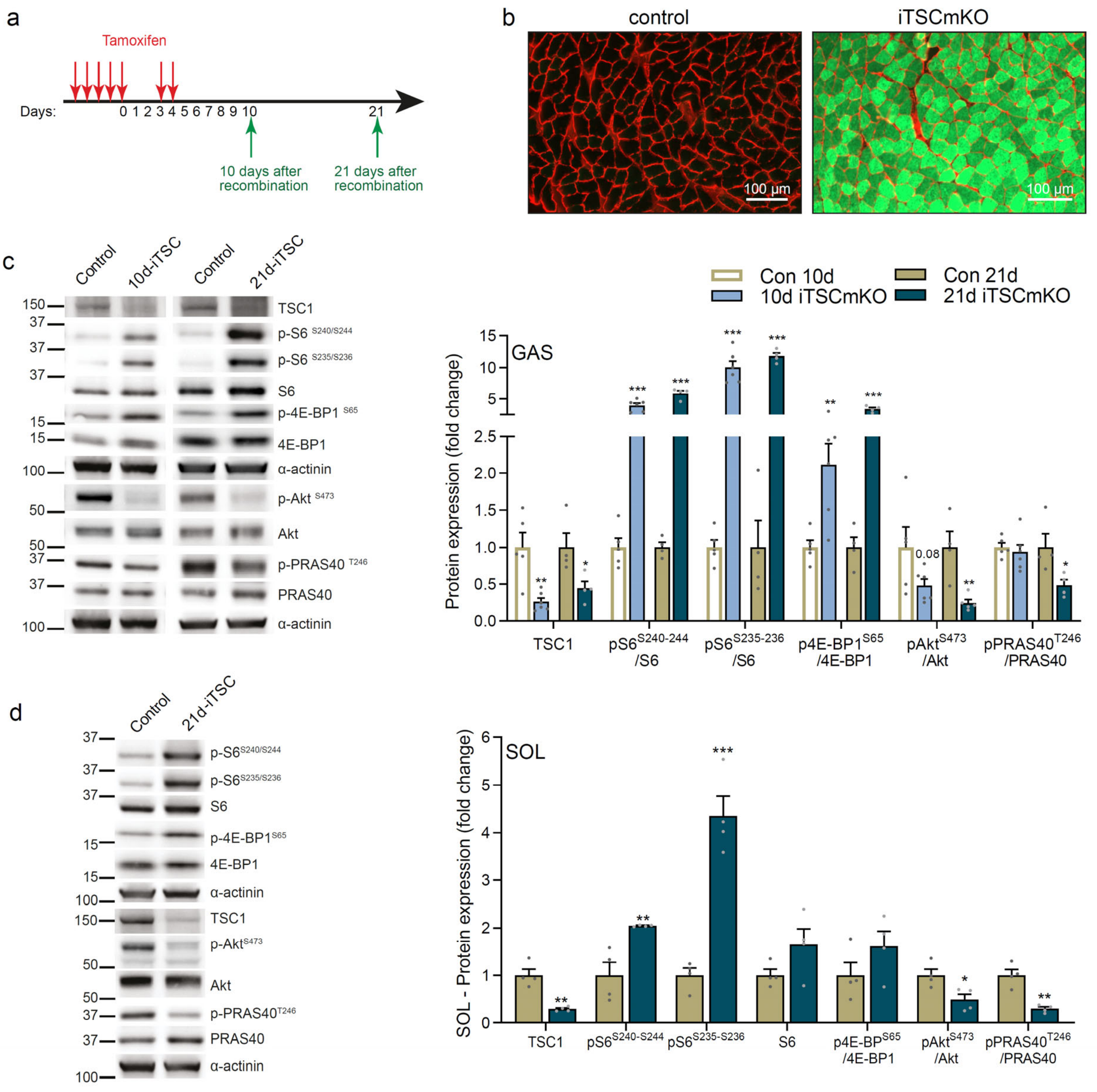

Figure S3. (a) tamoxifen treatment schedule for 10 day and 21 day induced TSC1 knockout mice (iTSCmKO). **(b)** Cross sections of *tibialis* anterior muscle from control and 10d iTSCmKO mice that also contain a *Rosa26* knock-in construct expressing enhanced green fluorescent protein (EGFP) as a Cre reporter. While control mice without HSA-MerCreMer do not express EGFP, all fibers of mice with Cre are EGFP positive. Muscle cross sections were also stained with antibodies against laminin (red). Western blots and quantification of phosphorylated and total proteins involved in the PKB/Akt-mTORC1 signaling pathway in **(c)** *gastrocnemius* muscle 10 days and 21 days after Tsc1 deletion and **(d)** *soleus* muscle 21 days after *Tsc1* deletion.

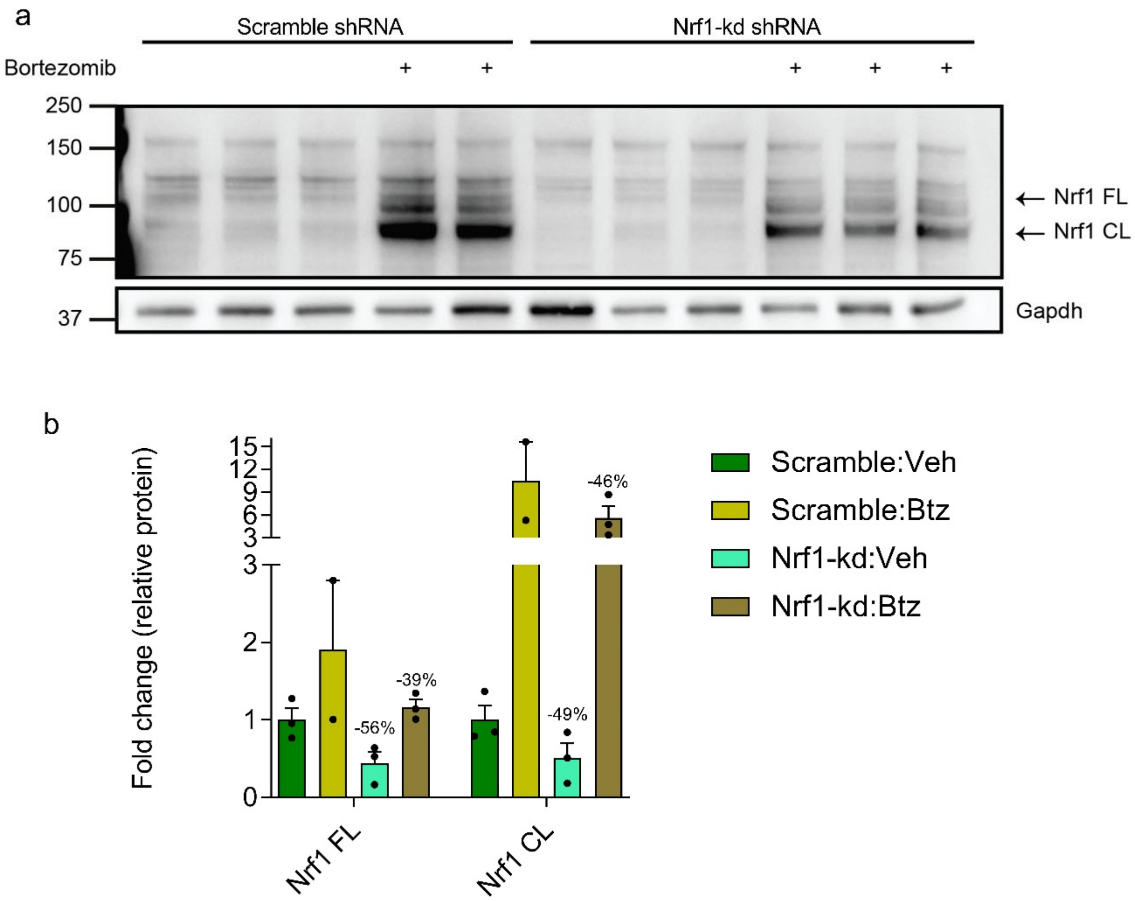

Figure S4. (a) Western blots and **(b)** quantification of cleaved (CL) and full length (FL) Nrf1 in mouse embryonic fibroblasts transfected with plasmids driving the expression of an shRNA directed to Nrf1 (Nrf1-kd) or to a scramble sequence (Scramble) and treated with Bortezomib (BTZ) for 4 hours prior to lysis. Data are presented as mean ± SEM. Percentage change from the respective scramble control is reported for each Nrf1-kd condition. Statistics were not performed due to low sample numbers.

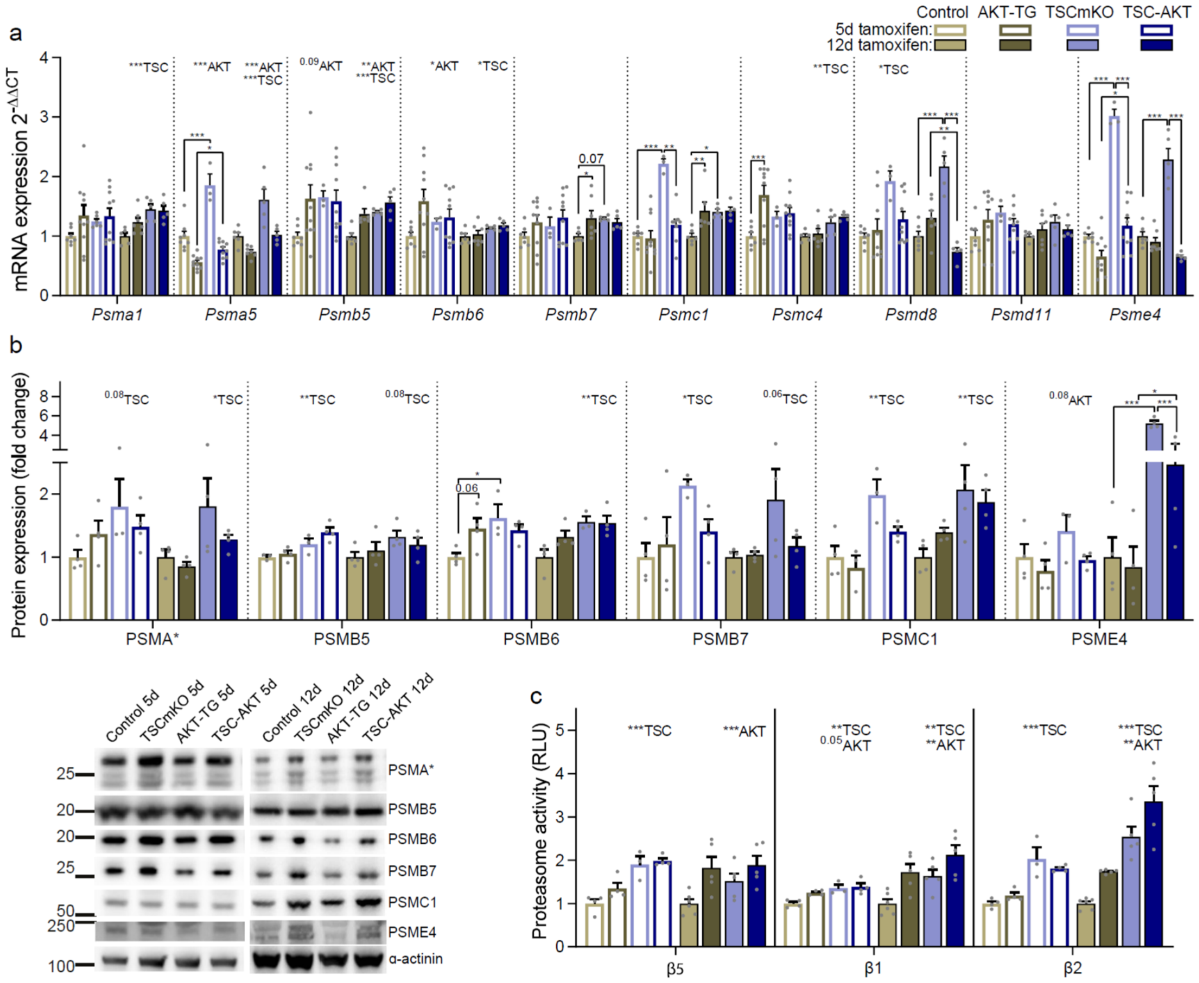

Figure S5. (a) mRNA (*gastrocnemius*) and **(b)** protein expression (*tibialis anterior*) of 26S proteasome subunits and the 20S activator PSME4 and **(c)** Luciferase-based peptidase activity (*plantaris*) of 20S proteasome catalytic enzymes in control, TSCmKO, AKT-TG and TSC-AKT mice treated with tamoxifen for 5 or 12 days. For (a), n=7 (5d Con), 6 (5d TSCmKO), 10 (5d AKT-TG and TSC-AKT), 5 (12d Con and TSCmKO), 6-7 (12d ATK-TG) and 5 (12d TSC-AKT). For (b), n=3-4. For (c), n=4 (5d Con, AKT-TG and TSC-AKT), 3 (5d TSCmKO), 6 (12d Con) and 5 (12d TSCmKO, AKT-TG and TSC-AKT). *Tubb* was used as the reference gene for (a), while α-actinin used as the protein loading control (b). Data are presented as mean ± SEM. Two way-ANOVAs with Sidak post hoc tests were used to compare data for each time point. *, **, and *** denote a significant difference between groups of $P < 0.05$, $P < 0.01$, and $P < 0.001$, respectively. For trends, where $0.05 < P < 0.10$, p values are reported.

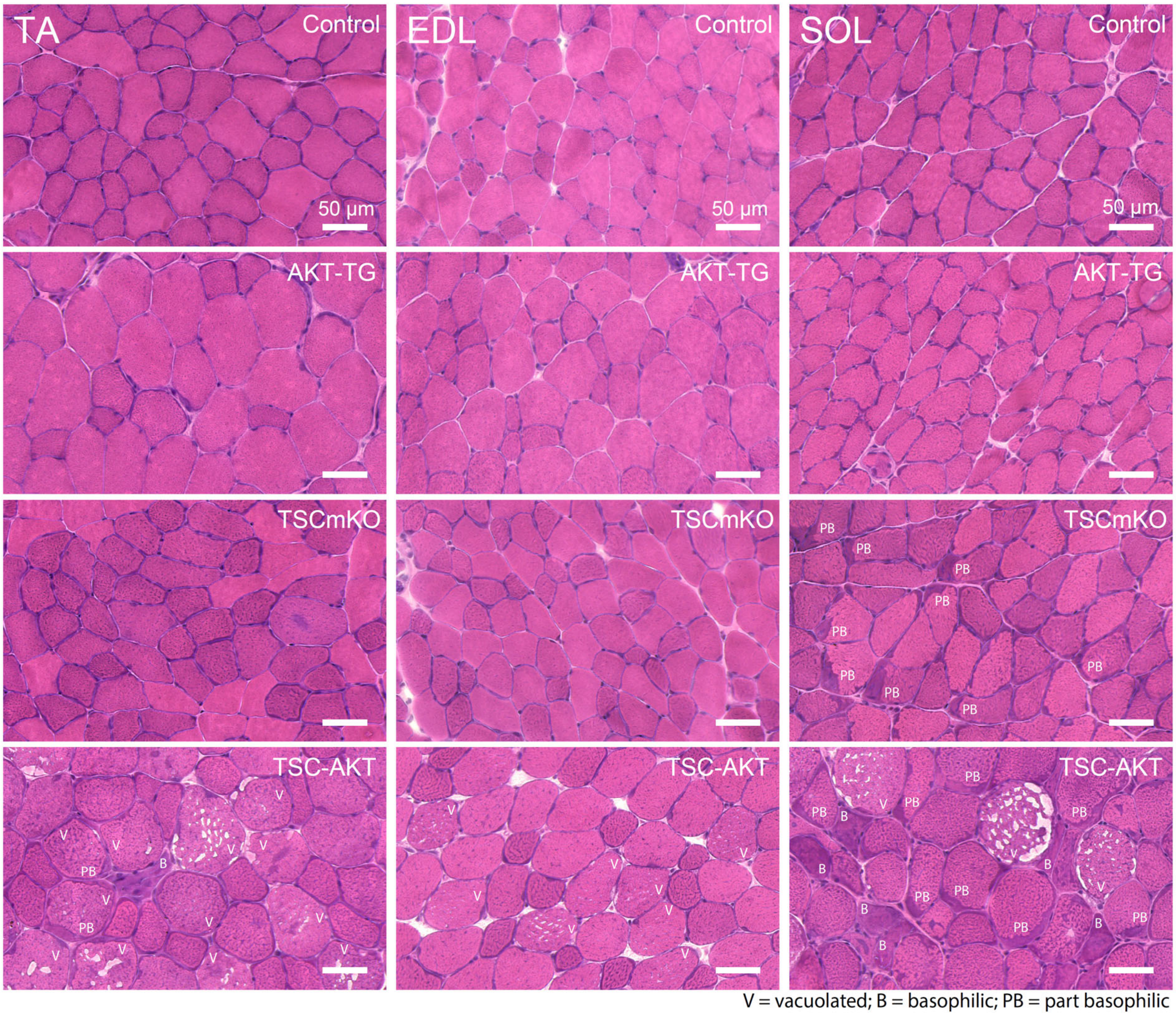

Figure S6. Representative images of Hematoxylin and eosin-stained cross sections of *tibialis anterior* (TA), *extensor digitorum longus* (EDL) and *soleus* (SOL) muscles collected from control, AKT-TG, TSCmKO and TSC-AKT mice. Vacuolated (V), basophilic (B) and partially basophilic (PB) fibers are indicated.

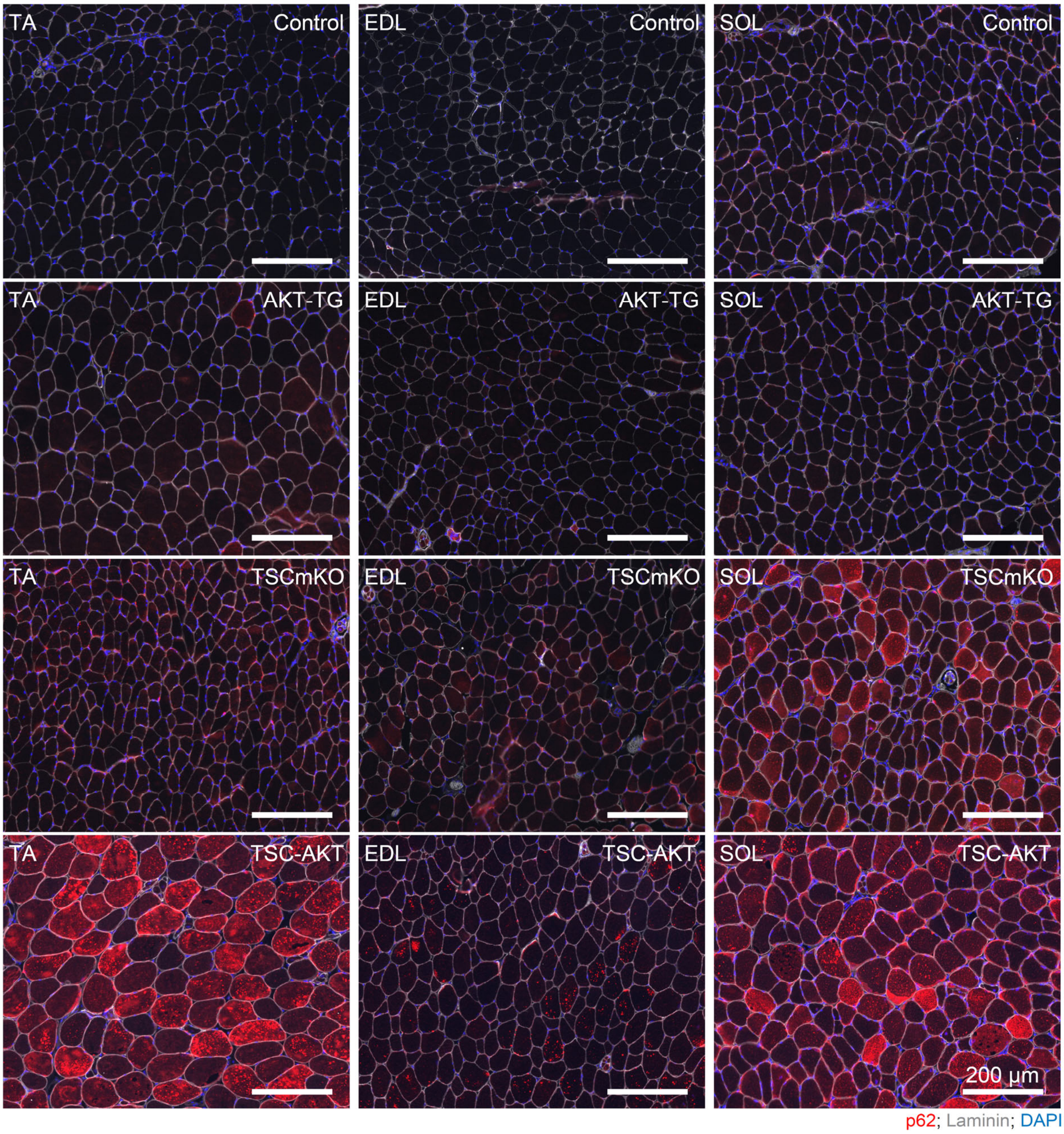

Figure S7. Representative images of *tibialis anterior* (TA), *extensor digitorum longus* (EDL) and soleus (SOL) muscle cross sections stained for p62 (red, quantified in Figure 7b), laminin (white), and DAPI (blue) in control, TSCmKO, AKT-TG and TSC-AKT mice after 20 days of tamoxifen treatment.

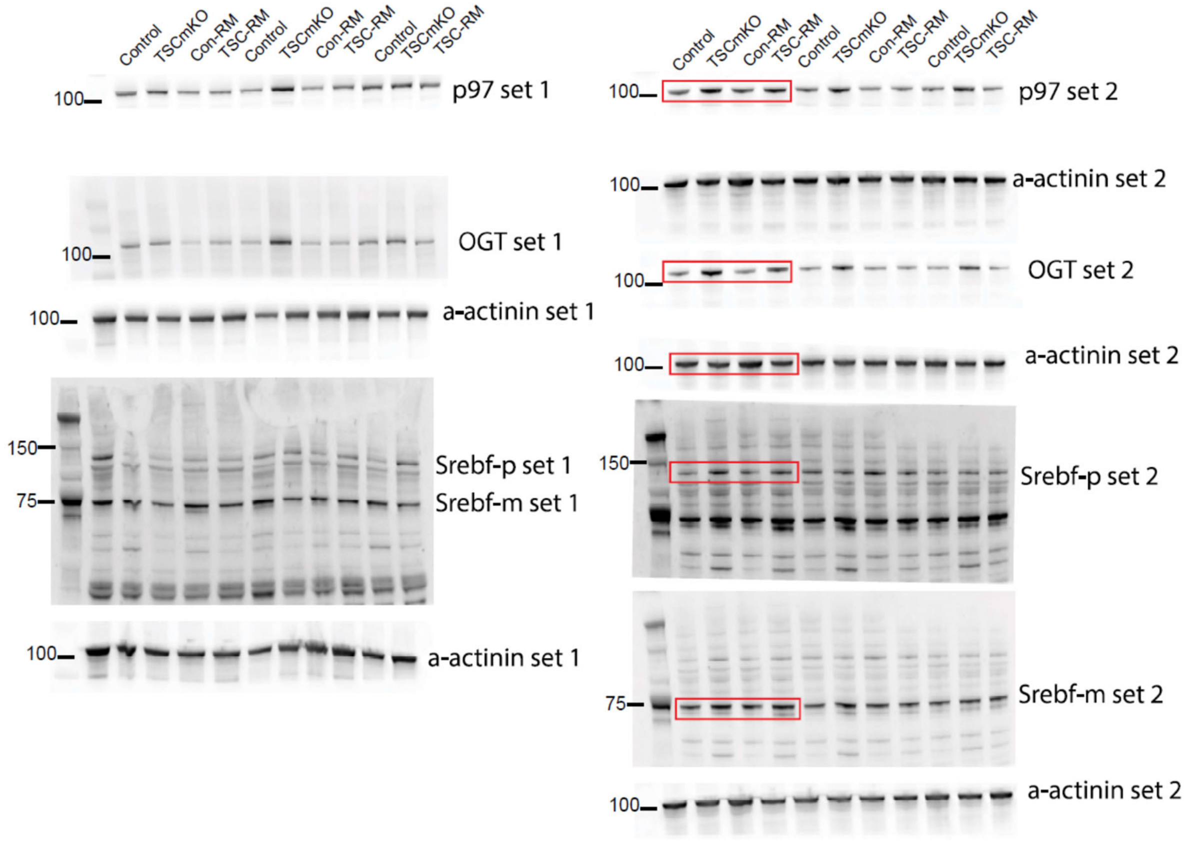

Figure S8. Uncropped Western blot images for Figure 1f. Red rectangles indicate cropped representative bands.

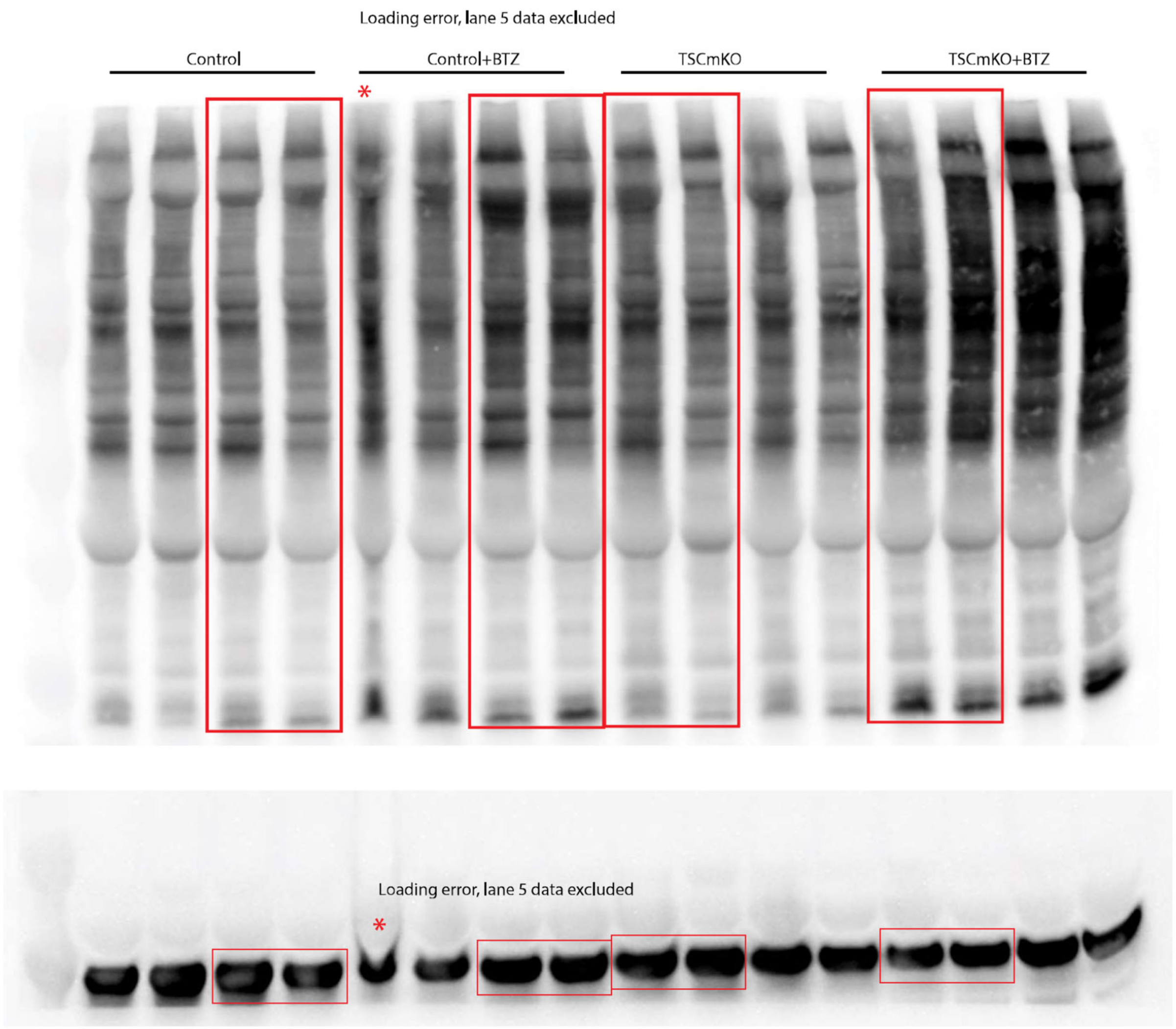

Figure S9. Uncropped Western blot images for Figure 1g. Red rectangles indicate cropped representative bands.

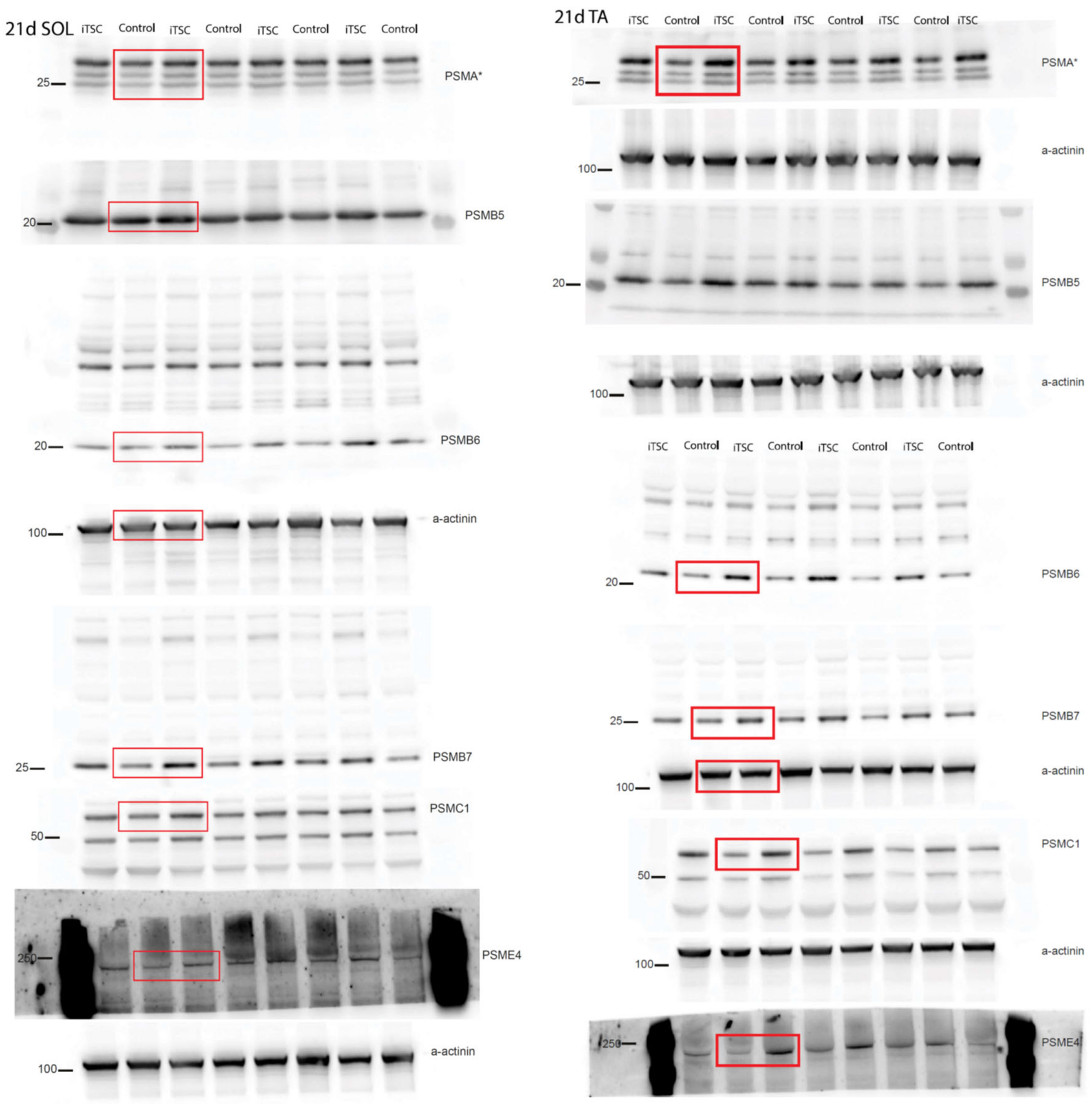

Figure S10. Uncropped Western blot images for Figure 2b. Red rectangles indicate cropped representative bands.

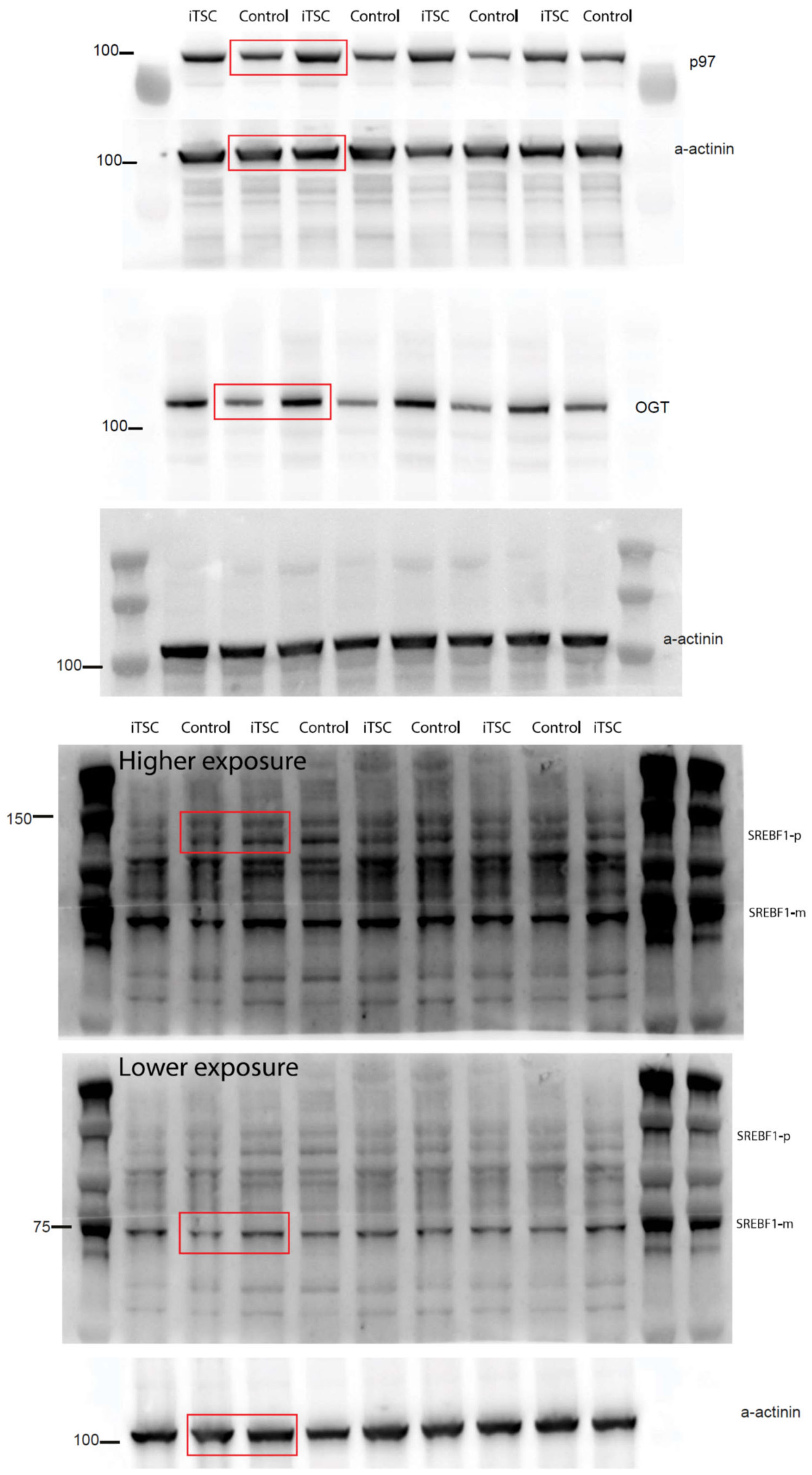

Figure S11. Uncropped Western blot images for Figure 2d. Red rectangles indicate cropped representative bands.

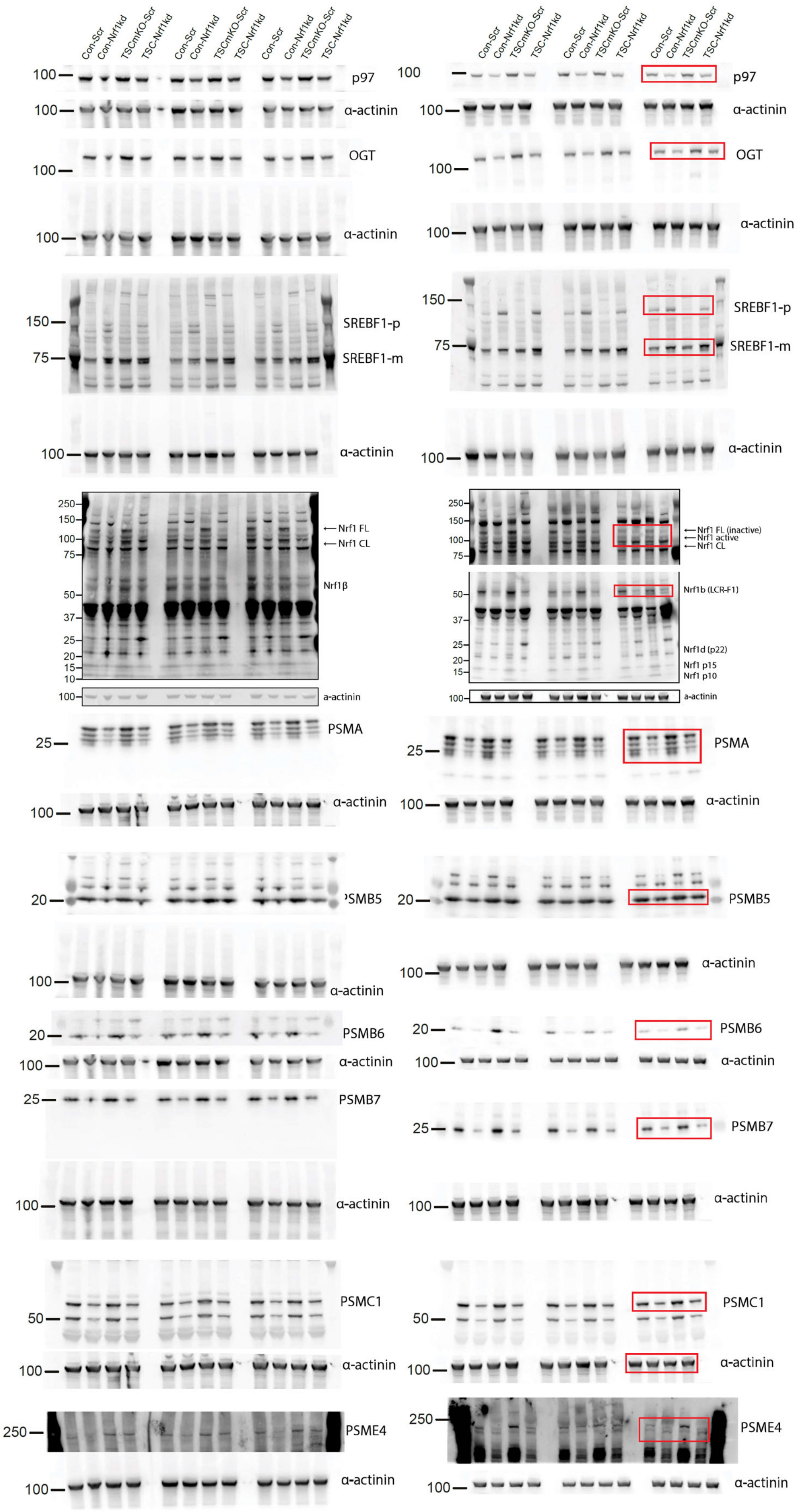

Figure S12. Uncropped Western blot images for Figure 3c. Red rectangles indicate cropped representative bands.

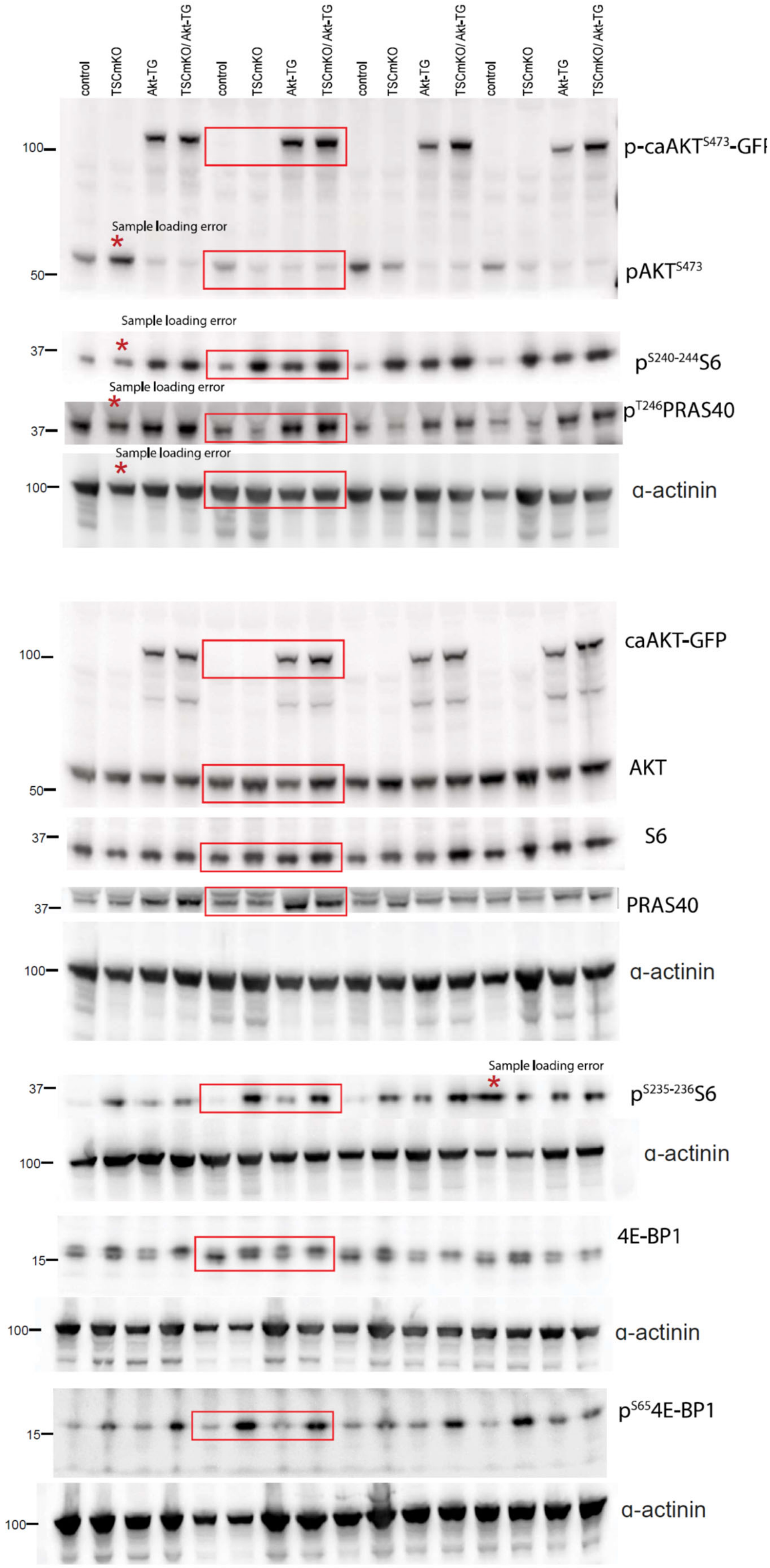

Figure S13. Uncropped Western blot images for Figure 4d. Red rectangles indicate cropped representative bands.

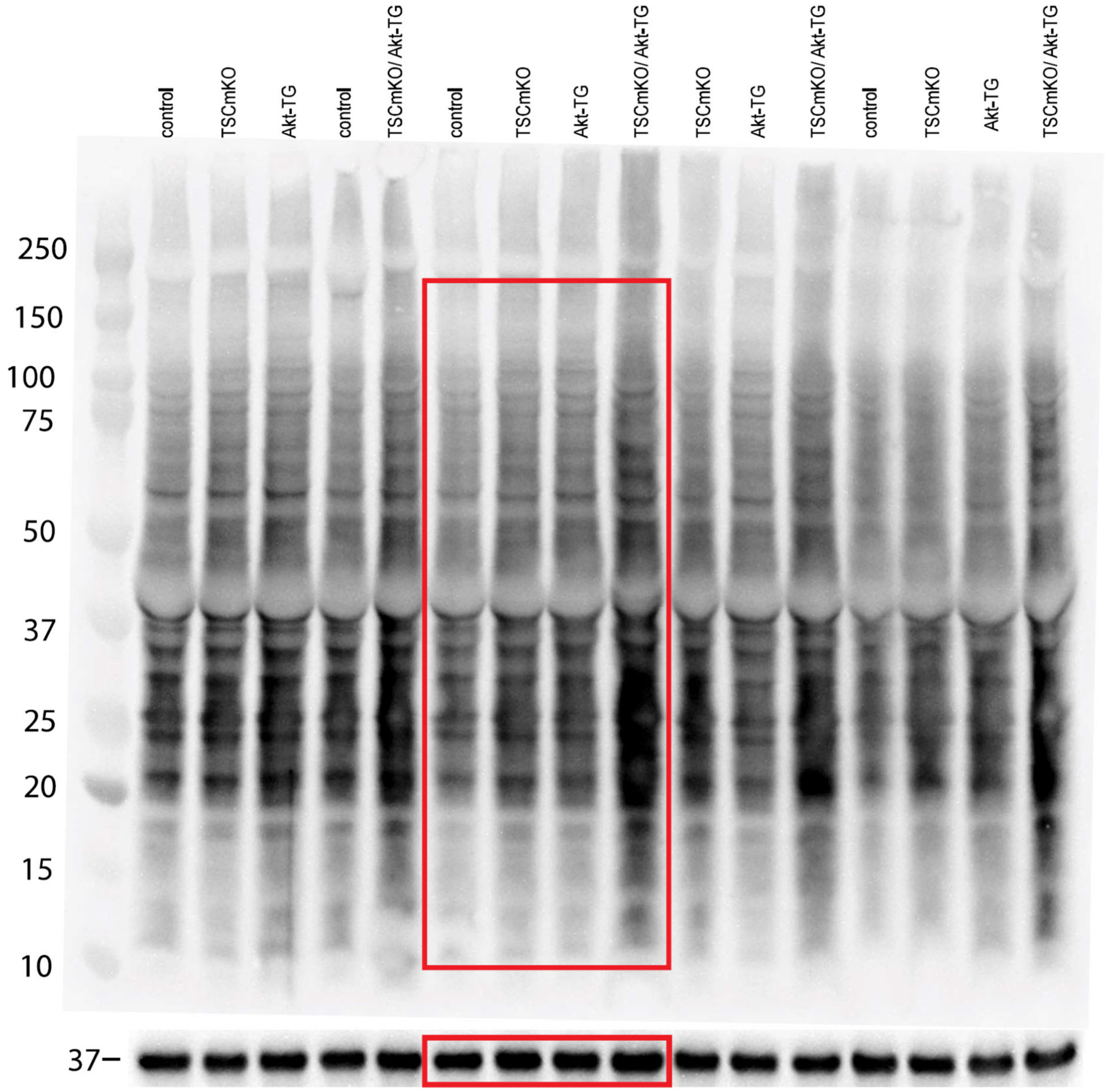

Figure S14. Uncropped Western blot images for Figure 4e. Red rectangles indicate cropped representative bands.

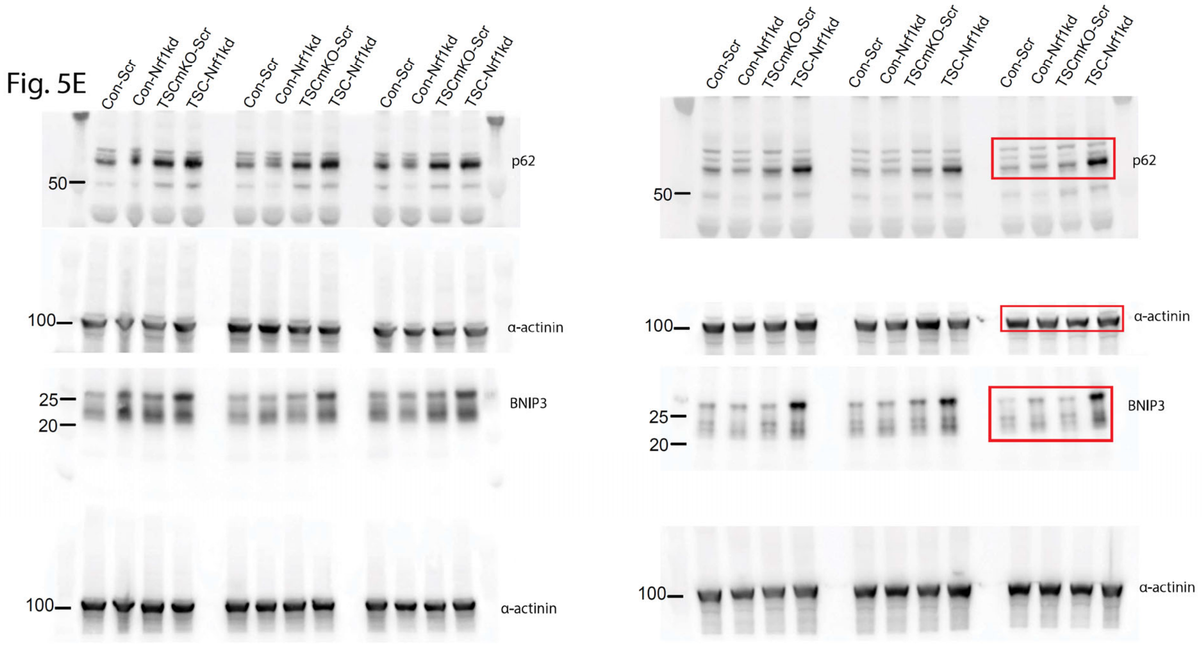

Figure S15. Uncropped Western blot images for Figure 5e. Red rectangles indicate cropped representative bands.

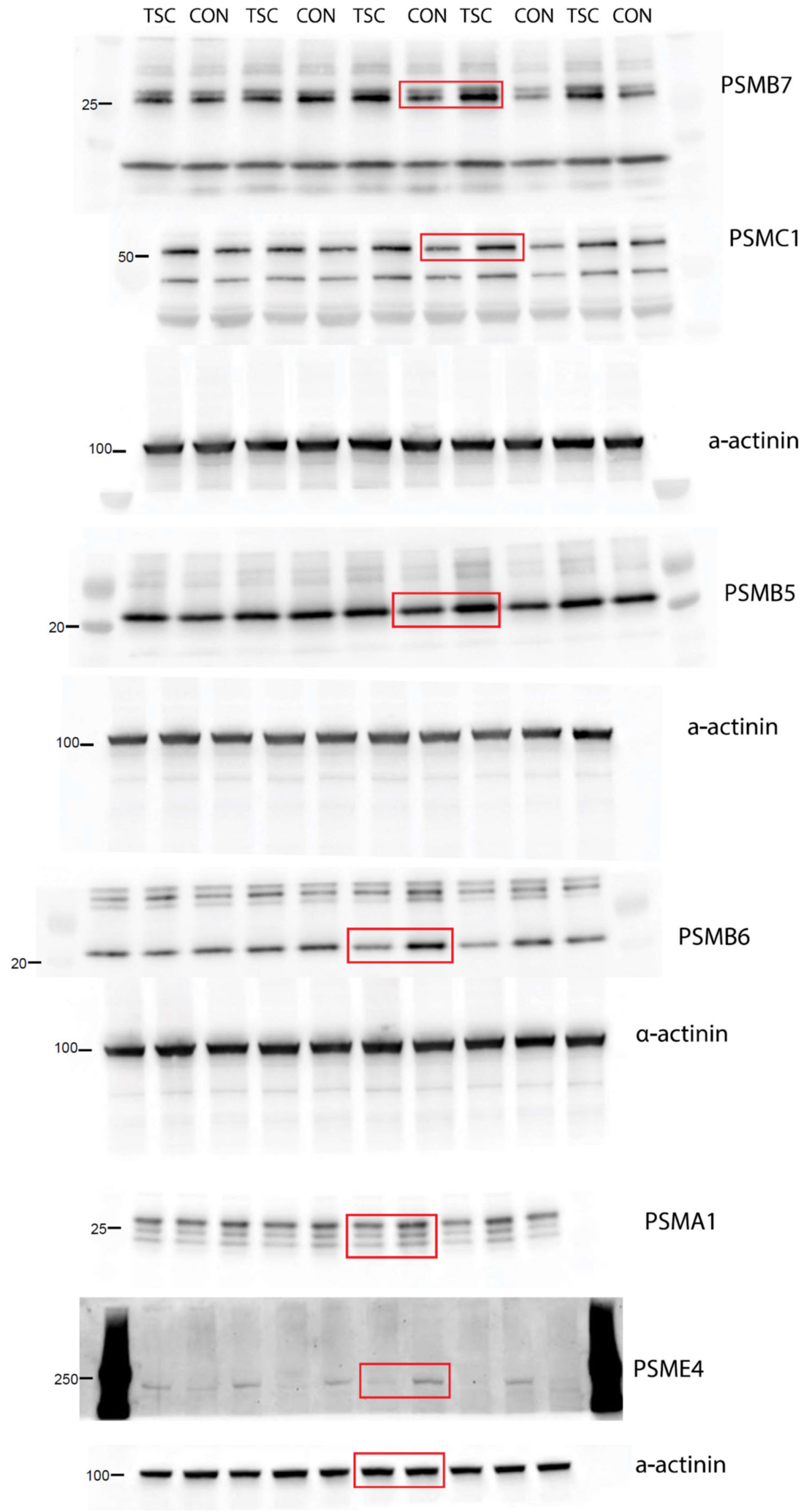

Figure S16. Uncropped Western blot images for Figure S1b. Red rectangles indicate cropped representative bands.

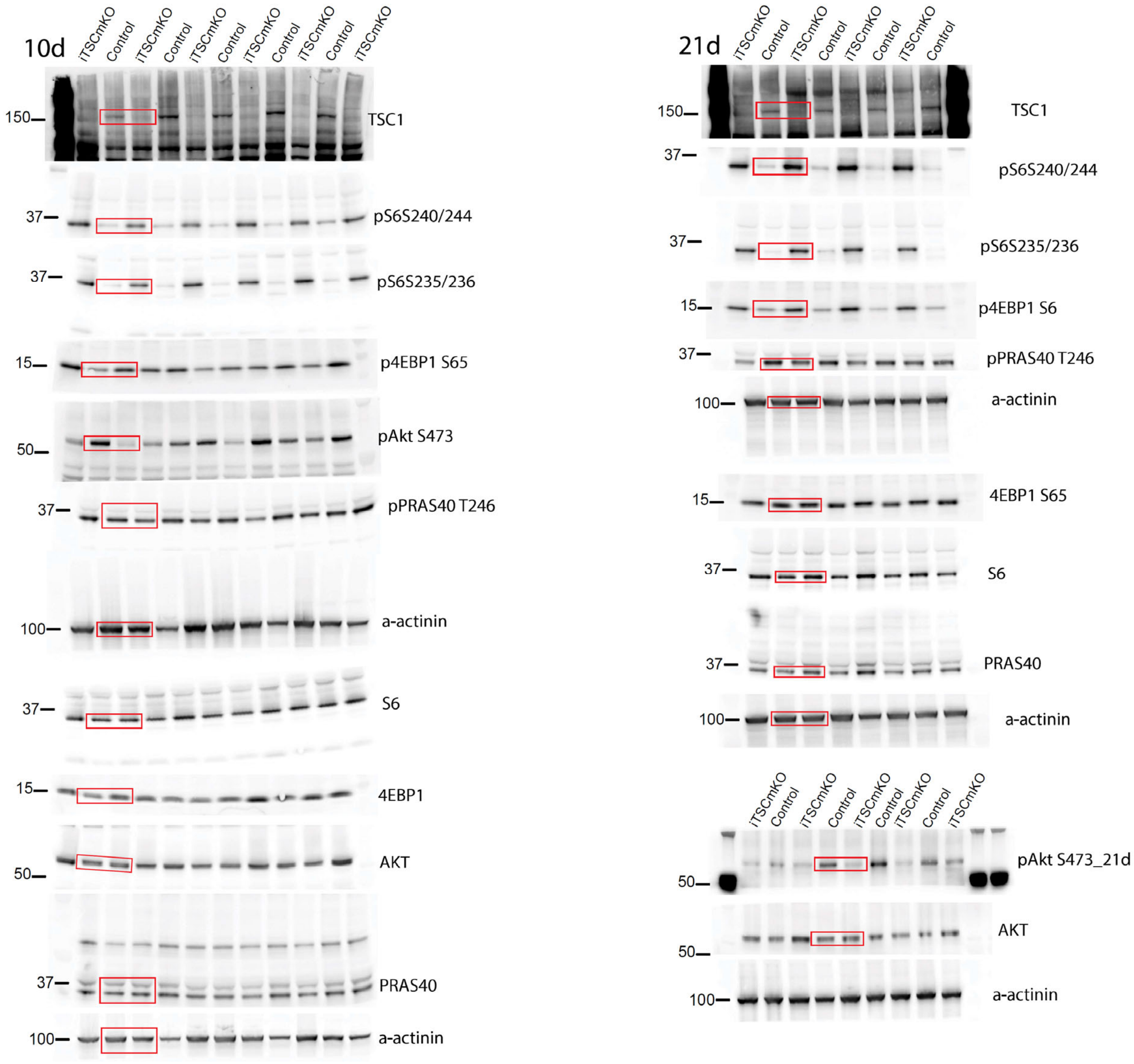

Figure S17. Uncropped Western blot images for Figure S3c. Red rectangles indicate cropped representative bands.

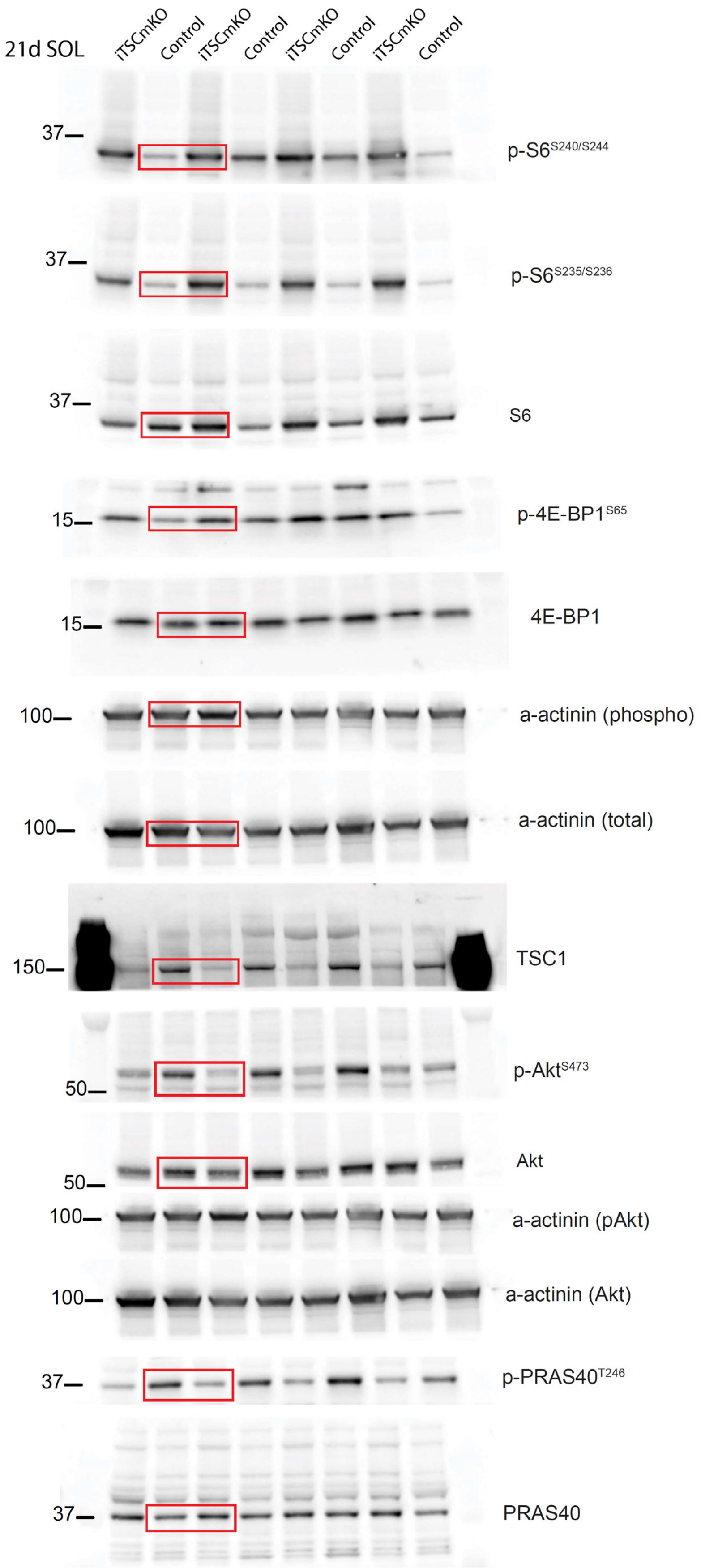

Figure S18. Uncropped Western blot images for Figure S3d. Red rectangles indicate cropped representative bands.

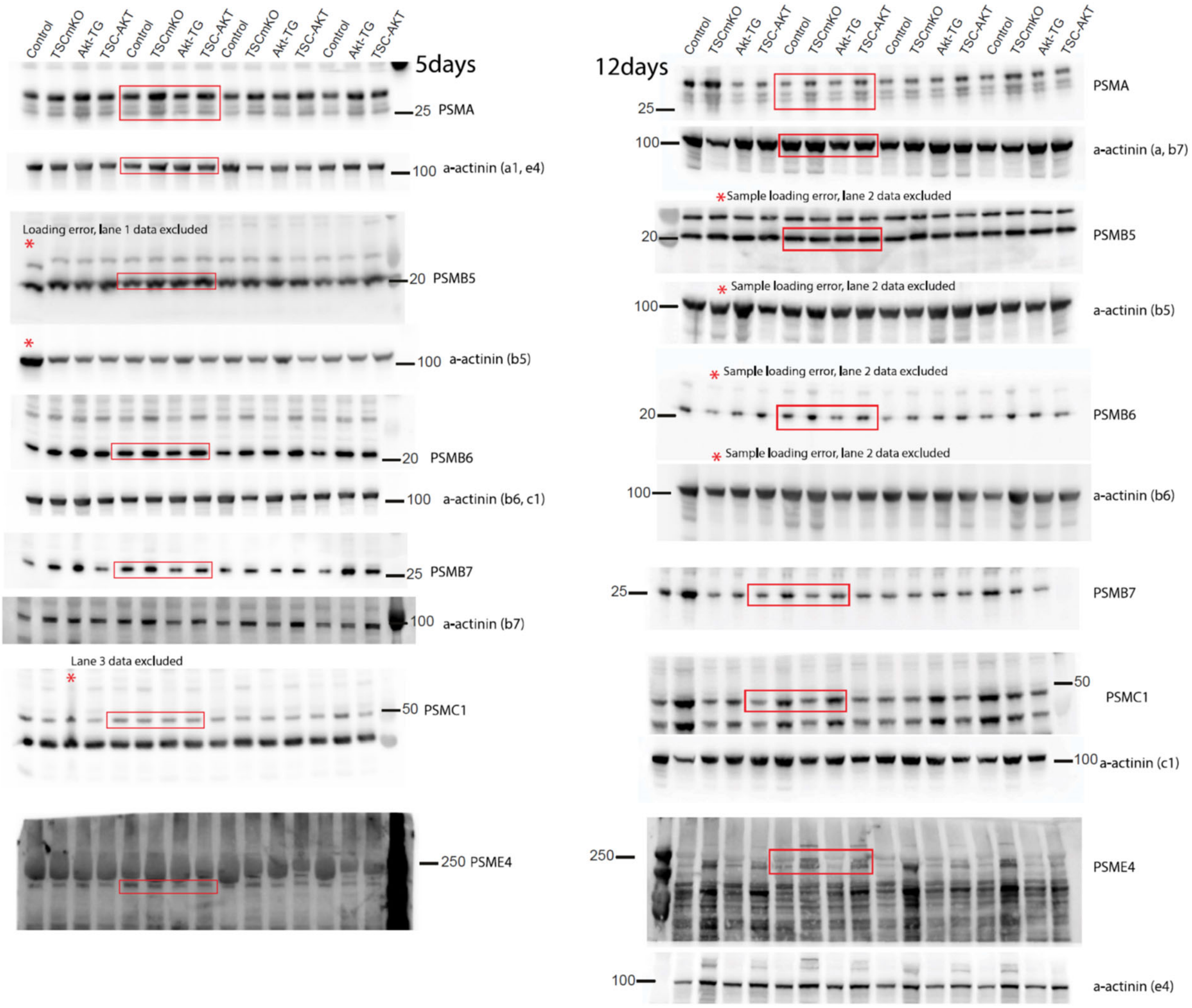

Figure S19. Uncropped Western blot images for Figure S5b. Red rectangles indicate cropped representative bands.

SUPPLEMENTARY TABLES

Table S1: Primers used for quantitative PCR analysis

Gene	Forward primer	Reverse primer
Actb	CAGCTTCTTTGCAGCTCCTT	GCAGCGATATCGTCATCCA
Bnip3	TTCCACTAGCACCTTCTGATGA	GAACACCGCATTTACAGAACAA
Tubb	GAAGCTGACCACACCCACCT	TGAAGAAGTGCAGGCGTGGG
Ctsd	CGTCTTGCTGCTCATTCTCGGCC	GCCGCCCACCTCCGTCATAGT
Ctsl	GTGGACTGTTCTCACGCTCA	TCCGTCCTTCGCTTCATAGG
Des	GAGGTTGTCAGCGAGGCTAC	CTTCAGGAGGCAGTGAGGAC
Fbxo30	TCGTGGAATGGTAATCTTGC	CCTCCCGTTTCTCTATCACG
Fbxo31	GTATGGCGTTTGTGAGAACC	AGCCCCAAAATGTGTCTGTA
Fbxo32	CTCTGTACCATGCCGTTCCT	GGCTGCTGAACAGATTCTCC
Gabarapl1	CATCGTGGAGAAGGCTCCTA	ATACAGCTGGCCCATGGTAG
Gadd45a	CCGAAAGGATGGACACGGTG	TTATCGGGGTCTACGTTGAGC
Map1lc3b	CACTGCTCTGTCTTGTGTAGGTTG	TCGTTGTGCCTTTATTAGTGCATC
Mdm2	GAGGATGATGAGGTCTATCG	GGAGGATTCATTTCATTGCAC
Trim63	ACCTGCTGGTGGAAAACA	AGGAGCAAGTAGGCACCTCA
Nfe2l1	CCCTACTCACCCAGTCAGTATG	CATCGTGCGAGGAATGAGGA
Nfe2l2	GCCCACATTCCCAAACAAGAT	CCAGAGAGCTATTGAGGGACTG
Nqo1	TTCTCTGGCCGATTCAGAG	GGCTGCTTGGAGCAAAATAG
Ogt	TTCGGGAATCACCCTACTTCA	TACCATCATCCGGGCTCAA
Ppp1r15a	GACCCCTCCAACTCTCCTTC	CTTCCTCAGCCTCAGCATTC
Psma1	CCTCAGGGCAGGATTCATCAA	GAGCGGCAAGCTCTGACTG
Psma5	AGCAATTGGCTCTGCTTCAG	GCATTCAGCTTCTCCTCCAT
Psmb5	AGGAGCCGCGAATCGAAATG	CCAGAAGGTACGGGTTGATCTC
Psmb6	CTGGGAAAACCGGGAAGTCTC	GAGTCCGCTCCTAGAACCAC
Psmb7	GTGTCGGTGTTTCAGCCAC	TCCGCTTCCAAGACAGCATT
Psmc1	AAGGGGGTCATTCTCTACGG	AAGCTCTGAGCCAACCACTC
Psmc4	TGGTCATCGGTCAGTTCTTG	CGGTCGATGGTACTCAGGAT
Psmd4	TCTCCTATTCTGGCTGGTGAA	CATGCTGCTTAGGTCTGGAAG
Psmd8	GCCTCAATCTCCTCTTCCTGCTATC	GTCTGTCATCTTTTTGGGTGTGC
Psmd11	AGGCAGACAGAAGCATTGAAA	GGTCCAAAATCCCATGAAACT
Psme4	AGCGTCAACAAGATAAGAATGCT	GCCCGATTCCTATATGCTCAAA
Sesn1	CCATAGGCCTTGGCTGATTA	AGCTGTGCTCCTCTGCTTTC
Sqstm1	AGGGAACACAGCAAGCTCAT	GACTCAGCTGTAGGGCAAGG
Traf6	GCAGTGAAAGATGACAGCGTGA	TCCCGTAAAGCCATCAAGCA
Ubb	GCTTACCATGCAACAAAACCT	CCAGTGGGCAGTGATGG
Ube4b	TGTCATCTTCCTTTCTTCTCTCT	TGGATTTTCATCTCGTGTCTG
Vcp	GGTTGGGGTTAGAGCAGCTT	GCGACTAATCAAACGACGGC

Table S2: Primary antibodies used for immunoblotting

Protein	Catalog number	Company	Dilution
4E-BP1	#9452	Cell Signaling Technology	1:1000
p-4E-BP1 S65	#9451	Cell Signaling Technology	1:1000
a-actinin	A7732	Sigma	1:5000
Akt	#9272	Cell Signaling Technology	1:1000
p-Akt S473	#9271	Cell Signaling Technology	1:1000
Bnip3	3769	Cell Signaling Technology	1:1000
GAPDH	#2118	Cell Signaling Technology	1:5000
Mono + Polyubiquitin	BML-PW8810	Enzo	1:500
Nfe2l1 (Nrf1)	#8052	Cell Signaling Technology	1:1000
Ogt	#24083	Cell Signaling Technology	1:1000
p62	GP62-C	Progen	1:1000
PRAS40	#2610	Cell Signaling Technology	1:1000
p- PRAS40 T246	#2997	Cell Signaling Technology	1:1000
PSMA*	BML-PW8195	Enzo	1:1000
PSMB5	ab3330	Abcam	1:1000
PSMB6	#13267	Cell Signaling Technology	1:1000
PSMB7	#13207	Cell Signaling Technology	1:1000
PSMB8	BML-PW8845	Enzo	1:1000
PSCM1	ab140450	Abcam	1:2000
PSMC5	ab140450	Abcam	1:2000
PSME4	18799-1-AP	Proteintech	1:500
Puromycin	MABE343	Millipore	1:5000
S6	#2217	Cell Signaling Technology	1:1000
p-S6 S235/S236	#2211	Cell Signaling Technology	1:1000
p-S6 S240/S244	#5364	Cell Signaling Technology	1:1000
SREBF1	Sc-8984	Santa Cruz	1:1000
TSC1	A300-316A	Bethyl	1:5000
Vcp (p97)	#2648	Cell Signaling Technology	1:1000
