# Supplementary material for: Dual roles of mTORC1-dependent activation of the ubiquitin-proteasome system in muscle proteostasis
Source: Commun Biol. 2022 Oct 27;5:1141. doi: 10.1038/s42003-022-04097-y (PMC9613904; doi:10.1038/s42003-022-04097-y)
Supplement: Supplementary file 2 — Description of Additional Supplementary Files [file 42003_2022_4097_MOESM2_ESM.pdf]

Description of Additional Supplementary Files

File name: Supplementary Data
Description: Source data for graphs and unsupervised list of atrophy-related genes.
