# Supplementary material for: Dual roles of mTORC1-dependent activation of the ubiquitin-proteasome system in muscle proteostasis
Source: Commun Biol. 2022 Oct 27;5:1141. doi: 10.1038/s42003-022-04097-y (PMC9613904; doi:10.1038/s42003-022-04097-y)
Supplement: Supplementary file 4 — Reporting summary [file 42003_2022_4097_MOESM4_ESM.pdf]

nature research

Corresponding author(s): Markus A. Rüegg, Daniel J. Ham, Giulia Milan.

Last updated by author(s): 30.09.2022

Reporting Summary

Nature Research wishes to improve the reproducibility of the work that we publish. This form provides structure for consistency and transparency in reporting. For further information on Nature Research policies, see our Editorial Policies and the Editorial Policy Checklist.

Statistics

For all statistical analyses, confirm that the following items are present in the figure legend, table legend, main text, or Methods section.

n/a	Confirmed	
☒	☐	The exact sample size (*n*) for each experimental group/condition, given as a discrete number and unit of measurement
☒	☐	A statement on whether measurements were taken from distinct samples or whether the same sample was measured repeatedly
☐	☒	The statistical test(s) used AND whether they are one- or two-sided<br>*Only common tests should be described solely by name; describe more complex techniques in the Methods section.*
☒	☐	A description of all covariates tested
☐	☒	A description of any assumptions or corrections, such as tests of normality and adjustment for multiple comparisons
☐	☒	A full description of the statistical parameters including central tendency (e.g. means) or other basic estimates (e.g. regression coefficient) AND variation (e.g. standard deviation) or associated estimates of uncertainty (e.g. confidence intervals)
☐	☒	For null hypothesis testing, the test statistic (e.g. F, t, r) with confidence intervals, effect sizes, degrees of freedom and P value noted<br>*Give P values as exact values whenever suitable.*
☒	☐	For Bayesian analysis, information on the choice of priors and Markov chain Monte Carlo settings
☒	☐	For hierarchical and complex designs, identification of the appropriate level for tests and full reporting of outcomes
☒	☐	Estimates of effect sizes (e.g. Cohen's *d*, Pearson's *r*), indicating how they were calculated

Our web collection on statistics for biologists contains articles on many of the points above.

Software and code

Policy information about availability of computer code

Data collection	RNAseq data were processed using Cutadapt v1.9.1 and Kallisto v0.43.1. Sequences of protein coding transcripts were selected based on genome assembly GRCm38 (release 92) and transcript annotations from Ensembl database. RNAseq data were analysed using the Bioconductor packages EdgeR v3.26.1 and RDAVIDWebService v1.22. SarcoAtlas (https://sarcoatlas.scicore.unibas.ch/) was developed using the R package Shiny v0.14.2. Mass-spectrometric data was statistically validated by the SafeQuant software tool
Data analysis	Data were analysed using GraphPad Prism 8.0.2.

For manuscripts utilizing custom algorithms or software that are central to the research but not yet described in published literature, software must be made available to editors and reviewers. We strongly encourage code deposition in a community repository (e.g. GitHub). See the Nature Research guidelines for submitting code & software for further information.

Data

Policy information about availability of data

All manuscripts must include a data availability statement. This statement should provide the following information, where applicable:

- Accession codes, unique identifiers, or web links for publicly available datasets
- A list of figures that have associated raw data
- A description of any restrictions on data availability

Raw and processed RNAseq data are available at Gene Expression Omnibus (GEO) under accession number GSE171322 and GSE139204. These data are also accessible using the web-based application, SarcoAtlas (https://sarcoatlas.scicore.unibas.ch/). Mass spectrometry proteomics data have been deposited to the ProteomeXchange Consortium via the PRIDE partner repository with the dataset identifier PXD034117 and 10.6019/PXD034117. Code is available upon request.

Field-specific reporting

Please select the one below that is the best fit for your research. If you are not sure, read the appropriate sections before making your selection.

☒ Life sciences ☐ Behavioural & social sciences ☐ Ecological, evolutionary & environmental sciences

For a reference copy of the document with all sections, see nature.com/documents/nr-reporting-summary-flat.pdf

Life sciences study design

All studies must disclose on these points even when the disclosure is negative.

Sample size	Sample sizes were based on previous experience in the lab (Castets, Cell Metabolism, 2013; Ham, Nature Communications, 2020).
Data exclusions	Data points were excluded only in the event of a clear technical error and are denoted in the supplementary data file.
Replication	Results were reproducible between experiments. Due to mouse availability and handling logistics, multiple sets of experimental groups were used, with comparable results between experiments. All attempts at replication were successful.
Randomization	Mice were assigned to groups based on genotype. Otherwise, they were matched for age, body mass and litter.
Blinding	In vitro measurements of muscle force were blinded for genotype. Due to technical/researcher limitations and obvious phenotypes, blinding was not performed for all other experiments.

Reporting for specific materials, systems and methods

We require information from authors about some types of materials, experimental systems and methods used in many studies. Here, indicate whether each material, system or method listed is relevant to your study. If you are not sure if a list item applies to your research, read the appropriate section before selecting a response.

Materials & experimental systems

n/a	Involved in the study	
☐	☒	Antibodies
☐	☒	Eukaryotic cell lines
☒	☐	Palaeontology and archaeology
☐	☒	Animals and other organisms
☒	☐	Human research participants
☒	☐	Clinical data
☒	☐	Dual use research of concern

Methods

n/a	Involved in the study	
☒	☐	ChIP-seq
☒	☐	Flow cytometry
☒	☐	MRI-based neuroimaging

Antibodies

Antibodies used

4E-BP1 #9452 Cell Signaling Technology; WB: 1:1000
p-4E-BP1 S65 #9451 Cell Signaling Technology; WB: 1:1000
a-actinin A7732 Sigma; WB: 1:5000
Akt #9272 Cell Signaling Technology; WB: 1:1000
p-Akt S473 #9271 Cell Signaling Technology; WB: 1:1000
Bnip3 3769 Cell Signaling Technology; WB: 1:1000
GAPDH #2118 Cell Signaling Technology; WB: 1:5000
Mono + Polyubiquitin BML-PW8810 Enzo; WB: 1:500
Nfe2l1 (Nrf1) #8052 Cell Signaling Technology; WB: 1:1000
Ogt #24083 Cell Signaling Technology; WB: 1:1000
p62 62-C Progen; IHC: 1:200; WB: 1:1000
PRAS40 #2610 Cell Signaling Technology; WB: 1:1000
p- PRAS40 T246 #2997 Cell Signaling Technology; WB: 1:1000
PSMA* BML-PW8195 Enzo; WB: 1:1000
PSMB5 ab3330 Abcam; WB: 1:1000
PSMB6 #13267 Cell Signaling Technology; WB: 1:1000
PSMB7 #13207 Cell Signaling Technology; WB: 1:1000
PSMB8 BML-PW8845 Enzo; WB: 1:1000
PSCM1 ab140450 Abcam; WB: 1:2000
PSMC5 ab178681 Abcam; WB: 1:2000
PSME4 18799-1-AP Proteintech; WB: 1:500
Puromycin MABE343 Millipore; WB: 1:5000
S6 #2217 Cell Signaling Technology; WB: 1:1000

p-S6 S235/S236 #2211 Cell Signaling Technology; WB: 1:1000
p-S6 S240/S244 #5364 Cell Signaling Technology; WB: 1:1000
SREBF1 Sc-8984 Santa Cruz; WB: 1:1000
TSC1 A300-316A Bethyl; WB: 1:5000
Vcp (p97) #2648 Cell Signaling Technology; WB: 1:1000
Myosin 7 (IHC: 1:50), DSHB BA-D5
Myosin 2 (IHC: 1:200), DSHB SC-71
Myosin 4 (IHC: 1:50), DSHB BF-F3
Laminin 2α (IHC: 1:100), Abcam 11576

Validation

p-S6S240/244 rabbit IgG monoclonal (WB: 1:1000), Cell Signaling 5364; https://www.cellsignal.com//products/primary-antibodies/phospho-s6-ribosomal-protein-ser240-244-d68f8-xp-rabbit-mab/5364
p-S6S235/236 (WB: 1:1000), Cell Signaling 2211; https://www.cellsignal.com//products/primary-antibodies/phospho-s6-ribosomal-protein-ser235-236-antibody/2211
S6 rabbit IgG monoclonal (WB: 1:1000), Cell Signaling 2217; https://www.cellsignal.com//products/primary-antibodies/s6-ribosomal-protein-5g10-rabbit-mab/2217
pS654EBP1 (WB: 1:1000), Cell Signaling 9451; https://www.cellsignal.com//products/primary-antibodies/phospho-4e-bp1-ser65-antibody/9451
4E-BP1 rabbit polyclonal (WB: 1:1000), Cell Signaling 9452; https://www.cellsignal.com//products/primary-antibodies/4e-bp1-antibody/9452
Bnip3 (WB: 1:1000), Cell Signaling 3769; https://www.cellsignal.com//products/primary-antibodies/bnip3-antibody-rodent-specific/3769
pT246PRAS40 (WB: 1:1000), Cell Signaling 2997; https://www.cellsignal.com//products/primary-antibodies/phospho-pras40-thr246-c77d7-rabbit-mab/2997
PRAS40 (WB: 1:1000), Cell Signaling 2610; https://www.cellsignal.com//products/primary-antibodies/pras40-antibody/2610
pS473AKT (WB: 1:1000), Cell Signaling 4058; https://www.cellsignal.com//products/primary-antibodies/phospho-akt-ser473-193h12-rabbit-mab/4058
Akt rabbit polyclonal (WB: 1:1000), Cell Signaling 9272; https://www.cellsignal.com//products/primary-antibodies/akt-antibody/9272
α-actinin mouse IgG1 EA-53 monoclonal (WB: 1:5000), Sigma A7732; https://www.sigmaaldrich.com/catalog/product/sigma/a7732
p62 guinea pig polyclonal (WB: 1:1000), Progen GP62-C; https://www.progen.com/anti-p62-sqstm1-c-terminus-guinea-pig-polyclonal-serum.html
Myosin 7 (IHC: 1:50), DSHB BA-D5; https://dshb.biology.uiowa.edu/BA-D5
Myosin 2 (IHC: 1:200), DSHB SC-71; https://dshb.biology.uiowa.edu/SC-71
Myosin 4 (IHC: 1:50), DSHB BF-F3; https://dshb.biology.uiowa.edu/BF-F3
Laminin 2α (IHC: 1:100), Abcam 11576 ; https://www.abcam.com/laminin-2-alpha-antibody-4h8-2-ab11576.html
GAPDH #2118 (WB: 1:5000) Cell Signaling Technology; https://www.cellsignal.com/products/primary-antibodies/gapdh-14c10-rabbit-mab/2118
Mono + Polyubiquitin (WB: 1:500) ML-PW8810 Enzo; https://www.enzolifesciences.com/ENZ-ABS840/mono-and-polyubiquitinylated-conjugates-recombinant-monoclonal-antibody-ubcj2/
Nfe2l1 (Nrf1) (WB: 1:1000) #8052 Cell Signaling Technology; https://www.cellsignal.com/products/primary-antibodies/tcf11-nrf1-d5b10-rabbit-mab/8052
Ogt (WB: 1:1000) 24083 Cell Signaling Technology; https://www.cellsignal.com/products/primary-antibodies/ogt-d1d8q-rabbit-mab/24083
PSMA* (WB: 1:1000) BML-PW8195 Enzo; https://www.enzolifesciences.com/BML-PW8195/proteasome-20s-alpha1-2-3-5-6-7-subunits-monoclonal-antibody-mcp231/
PSMB5 (WB: 1:1000) ab3330 Abcam; https://www.abcam.com/psmb5mb1-antibody-ab3330.html
PSMB6 (WB: 1:1000) #13267 Cell Signaling Technology; https://www.cellsignal.com/products/primary-antibodies/psmb6-e1k9o-rabbit-mab/13267?site-search-type=Products&N=4294956287&Ntt=13267+&fromPage=plp&_requestid=1102937
PSMB7 (WB: 1:1000) #13207 Cell Signaling Technology; https://www.cellsignal.com/products/primary-antibodies/psmb7-e1l5h-rabbit-mab/13207
PSMB8 (WB: 1:1000) BML-PW8845 Enzo; https://www.enzolifesciences.com/BML-PW8845/proteasome-20s-beta5i-subunit-monoclonal-antibody-lmp7-1/
PSCM1 (WB: 1:2000) ab140450 Abcam; https://www.abcam.com/proteasome-19s-s4psmc1-antibody-ab140450.html
PSMC5 (WB: 1:2000) ab178681 Abcam; https://www.abcam.com/psmc5-antibody-epr13565b-ab178681.html
PSME4 (WB: 1:500) 18799-1-AP Proteintech; https://www.ptglab.com/products/PSME4-Antibody-18799-1-AP.htm
Puromycin (WB: 1:5000) MABE343 Millipore; https://www.sigmaaldrich.com/CH/en/product/mm/mabe343?gclid=Cj0KCQjwqp-LBhDQARIsAO0a6aLloz7_hfWfyyN0mUzuKqQ4f3SzUTzBkvU7_mH3A2XY_6yuTGi-KugaAurxEALw_wcB
SREBF1 (WB: 1:1000) Sc-8984 Santa Cruz; https://www.scbt.com/p/srebp-1-antibody-h-160
TSC1 (WB: 1:5000) A300-316A Bethyl; https://www.bethyl.com/product/A300-316A/TSC1+Antibody
Vcp (p97) (WB: 1:1000) #2648 Cell Signaling Technology; https://www.cellsignal.com/products/primary-antibodies/vcp-antibody/2648

Eukaryotic cell lines

Policy information about cell lines

Cell line source(s)

SV40 immortalized mouse embryonic fibroblasts

Authentication

cell lines were not authenticated

Mycoplasma contamination

not tested for mycoplasm contamination

Commonly misidentified lines
(See ICLAC register)

N/A

Animals and other organisms

Policy information about studies involving animals; ARRIVE guidelines recommended for reporting animal research

Laboratory animals	TSCmKO, iTSCmKO, AKT-TG and TSC-AKT-TG mice (male and female) and WT controls were bred on a C57BL/6JRj background in house. Mice were kept on a 12 hr light-dark cycle (6 am to 6 pm) at 22°C (range 20-24°C) and 55% (range 45-65%) relative humidity.
Wild animals	This study did not involve wild animals
Field-collected samples	This study did not involve field-collected samples
Ethics oversight	All experiments were approved by the regional animal ethics Committee of Basel-Stadt, Switzerland.

Note that full information on the approval of the study protocol must also be provided in the manuscript.
